# RNA Surveillance Factor SMG5 Is Essential for Mouse Embryonic Stem Cell Differentiation

**DOI:** 10.3390/biom14081023

**Published:** 2024-08-17

**Authors:** Chengyan Chen, Yanling Wei, Xiaoning Jiang, Tangliang Li

**Affiliations:** 1 State Key Laboratory of Microbial Technology, Shandong University, Qingdao Campus, Qingdao 266237, China; 2 School of Basic Medical Sciences, Hangzhou Normal University, Hangzhou 311121, China

**Keywords:** nonsense-mediated mRNA decay, mouse embryonic stem cells, SMG5, c-MYC, differentiation

## Abstract

Nonsense-mediated mRNA decay (NMD) is a highly conserved post-transcriptional gene expression regulatory mechanism in eukaryotic cells. NMD eliminates aberrant mRNAs with premature termination codons to surveil transcriptome integrity. Furthermore, NMD fine-tunes gene expression by destabilizing RNAs with specific NMD features. Thus, by controlling the quality and quantity of the transcriptome, NMD plays a vital role in mammalian development, stress response, and tumorigenesis. Deficiencies of NMD factors result in early embryonic lethality, while the underlying mechanisms are poorly understood. SMG5 is a key NMD factor. In this study, we generated an *Smg5* conditional knockout mouse model and found that *Smg5*-null results in early embryonic lethality before E13.5. Furthermore, we produced multiple lines of *Smg5* knockout mouse embryonic stem cells (mESCs) and found that the deletion of *Smg5* in mESCs does not compromise cell viability. *Smg5*-null delays differentiation of mESCs. Mechanistically, our study reveals that the c-MYC protein, but not *c-Myc* mRNA, is upregulated in SMG5-deficient mESCs. The overproduction of c-MYC protein could be caused by enhanced protein synthesis upon SMG5 loss. Furthermore, SMG5-null results in dysregulation of alternative splicing on multiple stem cell differentiation regulators. Overall, our findings underscore the importance of SMG5-NMD in regulating mESC cell-state transition.

## 1. Introduction

In mammals, the embryonic development process is tightly controlled by temporal and spatial gene expression regulation, abnormalities of which can result in developmental defects and embryonic lethality [1,2,3]. Embryonic stem cells derived from the inner cell mass (ICM) of blastocysts at the E3.5 stage of mouse development are ideal cellular models to study gene expression regulation mechanisms during development. ESCs retain the ability to differentiate into three germ layers (the mesoderm, endoderm, and ectoderm), which will eventually develop into a complete organism. Understanding ESC cell fate determination, in terms of self-renewal and differentiation, is essential for utilizing the therapeutic potential of ESCs [4]. The transcriptional regulation and epigenetic modulation on gene expression in ESC self-renewal and differentiation regulation have been adequately studied [3,5,6,7], while post-transcriptional regulatory mechanisms in ESC fate determination are not well-defined. Recently, nonsense-mediated mRNA decay (NMD) has emerged as a critical regulatory pathway in ESC self-renewal and differentiation [8,9,10].

In eukaryotes, nonsense-mediated mRNA decay is a highly conserved post-transcriptional mechanism in gene expression [11,12,13]. NMD is originally identified to surveil transcriptome fidelity by degrading mRNAs with premature codons [11]. Furthermore, NMD destabilizes a subset of gene transcripts to fine-tune their protein outputs. The core components of NMD machinery, consisting of suppressors with morphogenetic effect on genitalia (SMGs, including SMG1, SMG5, SMG6, SMG7, SMG8, and SMG9), up-frameshift proteins (UPFs, including UPF1, UPF2, UPF3A, and UPF3B), eukaryotic release factors (eRF1 and eRF3) and an exon junction complex (EJC, including eIF4A3, RBM8A, MAGOH, and MLN51) were identified in yeasts and worms [14,15]. These factors orchestrate their functions in NMD target recognition, NMD machinery initiation, and degradation steps [11]. NMD is a translation-dependent mechanism. In the classical NMD pathway, prior to translation, EJCs are deposited at the boundary of exon junctions of mRNAs. Translating ribosomes move along the mRNAs until they encounter premature termination codons (PTCs). The NMD machinery takes action when the PTCs sit at least 50–55nt before the last exon junctions. The following steps include the forming of the SURF (SMG1–UPF1–eRF1–eRF3) complex and the DECID complex (decay-inducing complex). UPF1, phosphorylated by SMG1c, will recruit the SMG6 or SMG5/7 heterodimer to mediate mRNA decay steps. SMG6 possesses endoribonuclease activity and cleaves NMD targets at the vicinity of PTCs, while the SMG5/SMG7 heterodimer recruits de-capping and de-adenylation proteins to process the NMD targets. Recently, Boehm et al. showed that the endonuclease activity of SMG6 requires SMG5/7 heterodimer binding to NMD targets [16]. SMG5 KO causes higher magnitudes of NMD inhibition as compared with SMG6 KO and SMG7 KO [9,16]. Finally, all NMD targets will be degraded by the 5′-3′ exonuclease XRN1 and RNA exosome [11,12].

Knockdown of NMD core components in zebrafish causes embryonic lethality [17]. In mice, loss of NMD factors, including UPF1, UPF2, UPF3A, and SMG6, results in early embryonic lethality as well [8]. The NMD mutant embryos can survive to E3.5 and die before E9.5 [10], indicating NMD is essential for ESC differentiation and organogenesis [8]. This hypothesis is supported by several studies using human iPSCs and mouse ESCs [10,18,19]. Lou et al. discovered that the knockdown of UPF1 in P19, a mouse embryonic carcinoma cell line, leads to a decrease in transcripts of stemness genes and an increase in transcripts of neuronal lineage genes [18]. Furthermore, they observed that the downregulation of UPF1 in human embryonic stem cell H19 upregulates transcripts of *Smad2* and *Smad3*, which encode key components in the TGF-β signaling pathway. Conversely, the overexpression of UPF1 in hESCs upregulates target gene transcripts of the WNT and BMP signaling pathways, suggesting that NMD may regulate these pathways and facilitate mesoderm formation [19]. These findings indicated that UPF1-NMD promotes stemness. However, Li et al. showed that SMG6-null compromises mESC differentiation and demonstrated that SMG6 negatively modulates the expression of c-Myc by targeting the 3′UTR of the *c-Myc* gene, and thus influencing mESC differentiation [10]. mESCs with knockdown of *Smg1*, *Smg5*, *Upf1*, or *Upf2* using shRNAs displayed defective differentiation as well [10]. Interestingly, SMG6-null does not compromise ESC proliferation. Huth et al. used CRISPR-Cas9 technology and generated knockout ESCs of SMG5, SMG6, and SMG7 and further confirmed that NMD is dispensable for ESC proliferation [9]. Intriguingly, SMG5, SMG6, and SMG7 KO ESCs are all defective in differentiation. Mechanistically, they showed that, under the 2i condition, the PTC^+^ isoform of EIF4A2 is responsible for differentiation failure of SMG5 KO ESCs but not for SMG6 KO or SMG7 KO. These studies indicate that the NMD factor is dispensable for ESC self-renewal but essential for ESC differentiation [8,9,10]. However, these studies revealed that NMD factors may have different molecular targets during the ESC differentiation process.

The current study focused on the role of SMG5 in mouse embryonic development, self-renewal, and the differentiation of mouse embryonic stem cells. We conducted the gene targeting in mouse embryonic stem cells and generated *Smg5* conditional knockout mice. By crossing *Smg5* conditional knockout mice with the Nestin-Cre transgenic mouse line, we produced *Smg5* conventional knockout mice and found that SMG5 KO mice are embryonic lethal before E13.5. Furthermore, we generated multiple viable lines of SMG5-null mESCs and showed that SMG5 is essential for mESC differentiation. Our study highlights that SMG5 facilitates mESC differentiation by regulating c-MYC protein translation.

## 2. Materials and Methods

### 2.1. Mice and Genotyping Strategies

**Figure 1:**
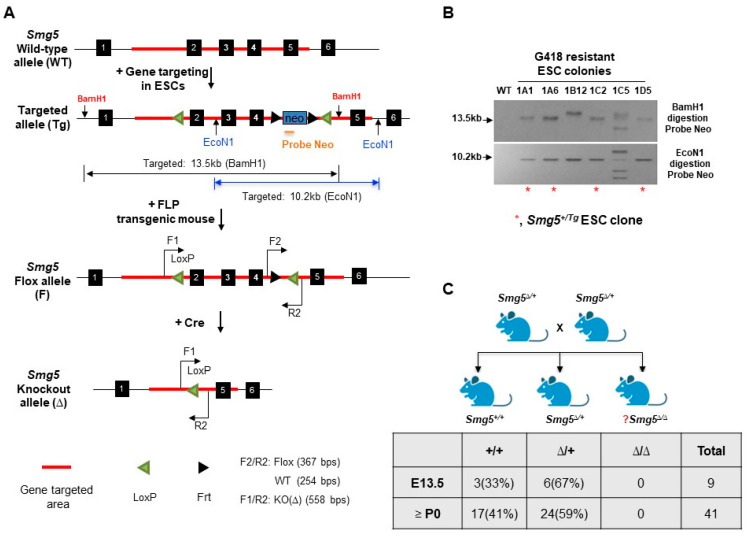
Generation of SMG5 inducible knockout mice. (**A**) Gene targeting strategy to generate *Smg5* conditional knockout mice (*Smg5^F/F^*). Wild-type allele (WT), gene-targeted allele (Tg), Flox allele (F), and knockout allele (Δ) are shown. Genotyping primers (F1, F2, and R2) are marked on the *Smg5* allele. (**B**) Southern blotting screening of mESC clone selected with G418 (Red asterisks indicate the correct gene-targeted ESC colonies). (**C**) Mating strategy to generate *Smg5*^Δ/Δ^ mice and genetic analysis showed that *Smg5*^Δ/Δ^ mice died before E13.5 (Note: P0, post-natal day 0). Original images can be found in Supplementary Materials.

In this study, the *Smg5* conditional knockout mouse (*Smg5*^F/F^) was generated by gene targeting in mouse embryonic stem cells with Cyagen Biosciences Inc. (Suzhou, China). In brief, the targeting vector was designed as follows: homology arms and cKO region were generated by PCR using BAC clone RP23-430O12 and RP23-132O9 from the C57BL/6J library as a template. Through the recombination strategy, 2 LoxP sites were inserted into intron 1 and intron 4. The Frt-Neo-Frt site, a screening marker for drug resistance in eukaryotic cells during gene targeting, was inserted between exon 4 and exon 5 of *Smg5* loci (Figure 1A). The DTA cassette placed upstream of the 5′ homology arm will be used for the negative selection. The gene-targeting vector was introduced into a mouse embryonic stem cell (C57BL/6N background) by electroporation, and G418 (275 μg/mL) was used for screening, selection, and expansion of mouse embryonic stem cell clones with G418 drug-resistance for Southern blotting identification. Hybridization of Southern blots was used on genomic DNAs from single ESC colonies to detect homologous recombination and single integration at the 5′ side was performed using BamHI restriction enzyme and Neo probe, which gave 13.5 kb (targeted allele, Tg) fragments. Southern hybridization on the 3′ side was performed by digestion DNA with the EcoNI restriction enzyme and Neo probe produced 10.2 kb (targeted allele, Tg). Finally, we identified a total of 4 positive gene target ESC clones (*Smg5*^+/Tg^) (Figure 1B). By injecting positive *Smg5*^+/Tg^ ESC clones into C57BL/6 blastocysts and subsequently transferring them into the uterus of wild-type mice, we produced *Smg5*^+/Tg^ chimeric mice and obtained germline offspring. Finally, *Smg5*^+/Tg^ mice were bred with FLP transgenic mice to remove neomycin cassettes, and thus *Smg5*^+/F^ mice were obtained. For routine genotyping of *Smg5* alleles, three primers were used, as follows: Smg5_F1: TTGTTCTTACCAGTGAGC, Smg5_R2: TGATGTGAGCATTGTCAG, Smg5_F2: GTTGAAGCTGTGACGTGG (WT allele: 254 bps; floxed allele: 367 bps; knockout allele (Δ): 558 bps).

To generate the *Smg5* conventional knockout allele, we crossed *Smg5*^F/F^ with Nestin-Cre transgenic mouse. Since Nestin-Cre transgenic mouse has leaky expression of Cre recombinase in male germlines [20], we were successful in obtaining the *Smg5* knockout allele (Δ) in the offspring (Supplementary Figure S1A,B). The *Smg5*^+/Δ^ mice were intercrossed to investigate the embryonic lethality of *Smg5*^Δ/Δ^ mice.

To generate SMG5 inducible knockout mice, *Smg5*^F/F^ mice were crossed with Cre-ER^T2+^ mice to produce *Smg5*^F/F^CreER^T2+^ mice (Supplementary Figure S1C) [21]. For genotyping of the Cre-ER^T2+^ transgene, a set of Cre-ER^T2^-specific primers (Cre-ErF: ATACCGGAGATCATGCAAGC; Cre-ErR: GATCTCCACCATGCCCTCTA) were used (PCR product of Cre-ER^T2+^ transgene: 552 bps).

All animals were maintained under specific pathogen-free conditions at the animal facility of Hangzhou Normal University, Hangzhou, China. Animal care and experiments were performed in accordance with the ethics committee guidelines of Hangzhou Normal University.

### 2.2. Generation of SMG5 Knockout mESC Lines

SMG5 inducible knockout mESCs (*Smg5*^F/F^Cre-ER^T2+^ mESCs) were generated and maintained by following a previously described protocol [21]. In brief, matings between male *Smg5*^F/F^Cre-ER^T2+^ mice and female *Smg5*^F/F^ mice were set up one day before vaginal plug checking (Supplementary Figure S1C). A clear vaginal plug marked the embryo stage as embryonic day 0.5 (E0.5). On E3.5, pregnant mothers were sacrificed, and blastocysts were flushed out. The blastocysts were further transferred into 24-well plates with mitomycin C-inactivated MEF feeders. After 4–5 days, small mESC clones were outgrown from blastocysts and further passaged to establish the final mESC lines. All the mESCs were maintained in ESC medium: DMEM (Gibco, Grand Island, NY, USA, 11995065) supplemented with 15% FCS (Gibco, Thornton, NSW, Australia, 10099141), 1× sodium pyruvate (Gibco, Grand Island, NY, USA, 11360070), 1× Pen/Strep (Gibco, Grand Island, NY, USA, 15140122), 1× GlutaMAX^TM^-1 (Gibco, Grand Island, NY, USA, 25050061), 1× non-essential amino acid (Millipore, Burlington, MA, USA, TMS-001-C), 1 μM 2-metacptoethanol (Gibco, Grand Island, NY, USA, 21985032), and 1000 units/mL LIF (Millipore, Billerica, MA, USA, ESG1107).

To produce SMG5-deficient mESC lines, two approaches were used in our study [10]. SMG5 inducible knockout mESCs (*Smg5*^F/F^Cre-ER^T2+^ mESCs) were treated with 4-OHT (1 μM, Sigma-Aldrich, Louis, MO, USA) for 5 consecutive days to induce excision of the exons 2–4 of *Smg5* loci. mESCs were further passaged for 2 times to establish *Smg5*-deficient mESC lines (***Smg5*^iKO^**). Alternatively, *Smg5*^F/F^CreER^T2-^ mESCs were transiently transfected with pCAG-GFP-Cre (#23776; Addgene, Cambridge, MA, USA). The GFP^+^ populations were sorted using FACS, and single-cell clones were isolated to establish *Smg5*-deficient mESCs (***Smg5*^Δ/Δ^**).

### 2.3. mESC Proliferation Assay

For the proliferation assay, the 1 × 10^6^ control or *Smg5*-deficient mESCs were plated in a 6-well plate precoated with the feeder layer. Every 3 days, mESC clones were dissociated with 0.25% trypsin-EDTA (Gibco, Grand Island, NY, USA, 25200056) and counted.

### 2.4. RNA Extraction and qPCR Analysis

Total RNA was extracted from mESCs using TRIzol^®^ Reagent (Sigma-Aldrich, Louis, MO, USA) and then reverse-transcribed into cDNA using Hifair™ III 1st Strand cDNA Synthesis SuperMix for qPCR (gDNA Digester Plus) (11141ES60, Yeasen, Shanghai, China). qPCR was carried out in a Step-One real-time PCR detection system (Bio-Rad, Hercules, CA, USA) using Hieff^®^ qPCR SYBR Green Master Mix (no Rox) (11201ES03, Yeasen, Shanghai, China). At least three independent samples were examined in triplicate for qPCR. The mRNA level of individual genes was calculated using the 2-ΔΔCq method and presented as relative (-fold) values of the control after being normalized with β-actin mRNA levels [22].

For NMD efficiency detection with qPCR, known NMD target genes were selected. qPCR primers for *Atf4*, *Gas5*, *Ddit3*, *Snhg16*, *Snhg12*, and *β-actin* were synthesized based on a previous publication [23]; primers for *Hnrnpl* and *Auf1* were obtained from Li et al. [10]. The gene-specific primers designed and used in this study are listed as follows:

*Smg5*: qF, GGAACTGCTGTGGAGAAAGG, qR, AGCGACCAGATGAGTCCTGT;
*Smg6*: qF, AAGGATTACATGCCCACCAG, qR, TCACAGGCACATTCCTTGTC;
*Smg7*: qF, AACCCAAATCGAAGTGAAGTCC, qR, ACACCGTACACAGTTCCTGTAA;
*Upf1*: qF, CTCGACGCACAAGTTGGAC, qR, TCCTCGAAGTTCAGCTCAGC;
*Upf2*: qF, GAAGAAGGAGGAGCTGAGACA, qR, CTCTTCCTCCTCCTCTCCCT;
*Smg1*: qF, GGGAAAGCTGTGCAAGAGAG, qR, CAACCTGCTCAGCAACTGAC.

For the identification of both normal and PTC^+^ isoforms through RT-PCR analysis, primers of *Hnrnphl*, *Trub2*, *Hnrnpk*, *Srsf6*, *Ptbp2*, *Pkm2*, *Jmjd6*, *Tmem183*, *Srsf3*, and *Rps9* were retrieved from a previous publication [24]. For the stemness and differentiation gene expression analysis, the qPCR primers were reported in a previous study [25].

### 2.5. Plasmid Construction and mESC Electroporation

cDNA of mouse *Smg5* was PCR amplified using the PrimeSTAR^®^ HS DNA polymerase (Takara, Tokyo, Japan, R040) with the following primer pairs: BspE1-Smg5-F, G***TCCGGA***ATGAGCCAAGGCCCTCC, SalI-Smg5-R, CG***GTCGAC***TCAACCAATCTCCTTCC. The PCR fragments and pEGFP-C1-EF1α [10] vector were subsequently digested using the restriction enzymes BspEI (New England Biolabs, Ipswich, MA, USA) and SalI (New England Biolabs, Ipswich, MA, USA), and further ligated using the T4 DNA ligase (New England Biolabs, Ipswich, MA, USA). The resulting plasmid (pEGFP-C1-EF1α-SMG5) was amplified in DH5α *E. Coli* and extracted with E.Z.N.A^®^Endo-free plasmid Midi kit (Omega BIO-TEK, Norcross, GA, USA). Finally, the plasmids (pEGFP-C1-EF1α and pEGFP-C1-EF1α-SMG5) were linearized with AseI (New England Biolabs, Ipswich, MA, USA) and electroporated into *Smg5*^Δ/Δ^ mESCs with the Bio-Rad Gene Pulser XCELL™ electroporator (Bio-Rad, Hercules, CA, USA). mESC clones with stable integration of EGFP- or EGPF-tagged SMG5 were selected with G418 (Gibco, 275 μg/mL, Grand Island, NY, USA).

### 2.6. Protein Extraction and Western Blotting Analysis

Total protein lysates were prepared from mESCs using RIPA buffer (Millipore, Billerica, MA, USA) supplemented with protease inhibitors and phosphatase inhibitors (Selleck, Houston, TX, USA). A total of 40–60 μg of total protein was separated with 4–20% gradient SDS-PAGE gels, then transferred onto PVDF membranes (Merck, Darmstadt, Germany). The PVDF membranes were further blocked for 1.5 h at room temperature with 5% non-fat dry milk in 1× Tris-buffered saline with Tween (1× TBST) buffer and then incubated overnight at 4 °C with primary antibodies. After removal of the primary antibodies, PVDF membranes were washed three times (10 min each time) in 1× TBST and incubated for 1 h at room temperature with secondary antibodies. The primary antibodies used in this study were as follows: Rabbit anti-SMG6 (1:1000, Ab87539, Abcam, Cambridge, UK); Rabbit anti-SMG7 (1:1000, A302-170A, Bethyl, Montgomery, TE, USA); Rabbit anti-SMG5 (1:500, Ab33033, Abcam, Cambridge, UK); Rabbit anti-OCT4 (1:1000, 2840S, Cell Signaling Technology, Danvers, MA, USA); Rabbit anti-c-MYC (1:1000, 5605S, Cell Signaling Technology, Danvers, MA, USA); Rabbit anti-SOX2 (1:1000, 23064S, Cell Signaling Technology, Danvers, MA, USA); Rabbit anti-NANOG (1:1000, 8822S, Cell Signaling Technology, Danvers, MA, USA); Mouse anti-β-actin (1:5000, A5441, Sigma-Aldrich, Burlington, MA, USA); and Mouse anti-LaminB1 (1:3000, sc-374015, Santa Cruz, Idaho, USA). The secondary antibodies were HPR-Conjugated Goat Anti-Mouse IgG (1:5000, AP124P, EMD Millipore, Burlington, MA, USA) and HPR-Conjugated Goat Anti-Rabbit IgG (1:5000, AP132P, EMD Millipore, Burlington, MA, USA).

### 2.7. In Vitro Differentiation Assay

The in vitro differentiation of mESCs was conducted following the previously described protocol [10]. In brief, 2 × 10^5^ control and *Smg5* deficient mESCs were plated in gelatin-coated culture dishes and cultured in differentiation medium (ESC medium without LIF). The fresh differentiation medium was replaced every 3 days. After 4 days, cells in differentiation cultures were collected for AP staining, qPCR, or Western blotting. For AP staining, cells were fixed with 4% PFA for 10 min at room temperature. Then, an AP staining kit (Sigma-Aldrich, Louis, MO, USA) was used to investigate the differentiation status. The images were captured with OLYMPUS cellSens software (Standard Version) on an Olympus microscope BX53 installed with a DP80 camera (Olympus, Shinjuku City, Tokyo, Japan).

### 2.8. mRNA Stability Assay

The half-life of mRNAs was determined by following a previously published protocol [26,27]. In brief, 1 × 10^6^ mESCs were grown on gelatin-coated cell culture dishes. The mESCs were treated with actinomycin D (Sigma-Aldrich, Louis, MO, USA, A4262, 10 μg/mL). The mESC samples were harvested at time points of 0 h, 1 h, 2 h, and 3 h. Total RNAs were extracted with Trizol and further processed with first-strand synthesis with Hifair™ III 1st Strand cDNA Synthesis SuperMix for qPCR (gDNA Digester Plus) (11141ES60, Yeasen, Shanghai, China). qPCR was performed to reveal the expression of indicated genes. mRNA half-life was determined by fitting an exponential decay curve to the relative expression at each time point, and t_1/2_ was calculated based on the average expression at each time point. The mean t_1/2_ for each condition is represented [28]. The expression of genes at time 0 was denoted as 100%.

### 2.9. Protein Synthesis Analysis

Surface sensing of translation assay (SUnSET) was used to investigate global protein synthesis [29]. In brief, control and *Smg5*-deficient mESCs were treated with 10 μg/mL puromycin (Sigma-Aldrich, Louis, MO, USA, P8833) for 1 h before harvest. As a positive control for protein synthesis inhibition, Cycloheximide (CHX, 100 μM, Selleck, Houston, TX, USA, S7418) was added into the ESC culture for 10 min before puromycin treatment. A puromycin-specific antibody (1:1000, Sigma-Aldrich, Louis, MO, USA, ZMS1016) was used to determine the global protein synthesis in the control and *Smg5*-deficient mESCs.

### 2.10. RNA-Seq and Data Analysis

Total RNAs were isolated from mESCs using TRIzol^®^ Reagent (Sigma-Aldrich, Louis, MO, USA, T9424). The RNA quality was measured with the Bioanalyzer 2100 system (Agilent Technologies, Santa Clara, CA, USA). All the samples displayed a RIN (RNA integrity number) higher than 9. Then, the mRNA, which was purified from total RNAs using poly-T-oligo-attached magnetic beads, was utilized for cDNA library preparation. The cDNA library preparation was assessed on the Agilent Bioanalyzer 2100 system. Raw data (raw reads) were processed and calculated. All subsequent analyses were based on high-quality data. DEGs of the control and *Smg5*-deficient mESCs (three biological replicates per condition) were analyzed using the DESeq2 R package (1.20.0). The resulting *p*-values were adjusted using Benjamini and Hochberg’s approach for controlling the false discovery rate. Genes with an adjusted *p*-value (Padj) < 0.05 found by DESeq2 were assigned as differentially expressed. The clusterProfiler R package was used to test the statistical enrichment of differential expression genes in Gene Ontology (GO).

### 2.11. Statistical Analysis

Values are presented as means ± standard deviation (S.D.). For determination of the statistical significance between two groups, the unpaired Student’s *t*-test was performed. Statistical significance was expressed as *p* < 0.05, *; *p* < 0.01, **; and *p* < 0.001, ***. The statistical analysis in this study was performed with GraphPad Prism 9.0.0 (GraphPad Software, San Diego, CA, USA).

## 3. Results

### 3.1. SMG5 Deletion Causes Earlier Embryonic Lethality

To investigate the biological function of SMG5 during mouse embryonic development, we crossed the *Smg5*^F/F^ mice with the Nestin-Cre transgenic mice (Supplementary Figure S1A). Nestin-Cre transgenic mice were extensively used to study the function of the GOI (gene of interest) in neural stem cells and brain development. However, Nestin-Cre mice have leaky expression of Cre recombinase in testis (Supplementary Figure S1B) [20]. By crossing *Smg5*^F/+^ Nestin-Cre^+^ males with wild-type females, *Smg5*^+/Δ^ mice were produced. When *Smg5*^+/Δ^ mice were intercrossed, no viable *Smg5*^Δ/Δ^ newborns were produced. By E13.5, *Smg5*^Δ/Δ^ embryos were not present (Figure 1C). These findings indicate that SMG5 is essential for embryonic development in mice.

### 3.2. Generation and Characterization of Smg5 Knockout mESCs

**Figure 2:**
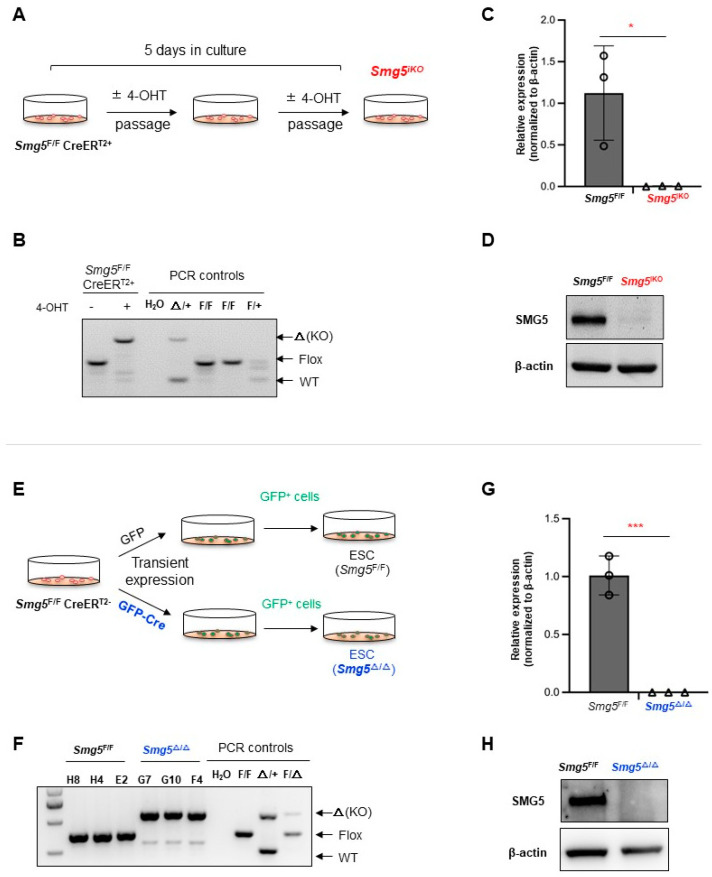
Establishment of *Smg5* deficient mESCs. (**A**) Strategy for establishing *Smg5*^iKO^ mESC lines by 4-OHT induction in *Smg5*^F/F^CreER^T2+^ mESCs. (**B**) PCR analysis on *Smg5* locus (exons 2–4) deletion in *Smg5*^F/F^CreER^T2+^ mESCs after 4-OHT treatment for 5 days. (**C**) qPCR analysis on *Smg5* mRNA expression in *Smg5*^F/F^CreER^T2+^ mESCs after 4-OHT treatment for 5 days. (**D**) Western blotting analysis on SMG5 protein expression in *Smg5*^F/F^CreER^T2+^ mESCs after 4-OHT treatment for 5 days. β-actin is used as a loading control. (**E**) Strategy to establish *Smg5*^Δ/Δ^ mESC lines by transfecting pCAG-GFP-Cre into *Smg5*^F/F^CreER^T2-^ mESCs. (**F**) PCR analysis on *Smg5* locus (exons 2-4) deletion in *Smg5*^Δ/Δ^ mESCs. (**G**) qPCR analysis on *Smg5* mRNA expression in *Smg5*^Δ/Δ^ mESCs. (**H**) Western blotting analysis on SMG5 protein expression in *Smg5*^Δ/Δ^ mESCs. β-actin is used as a loading control. Note: unpaired Student’s *t*-test was carried out for statistical analysis. *, *p* < 0.05; ***, *p* < 0.001. Original images can be found in Supplementary Materials.

In order to study the function of SMG5 in mouse ESCs, we first generated multiple lines of *Smg5* inducible knockout mESCs (*Smg5*^F/F^CreER^T2+^). We utilized two strategies to obtain the *Smg5*-deficient mESCs (Figure 2). First, multiple lines of *Smg5* knockout mESCs were established by treating *Smg5*^F/F^CreER^T2+^ mESCs with 4-OHT for 5 days (Figure 2A). The deletion of SMG5 was confirmed by normal PCR genotyping (Figure 2B), qPCR, and Western blotting (Figure 2C,D). In these *Smg5*-deficient mESCs, SMG5 was completely absent at the mRNA level and protein level (Figure 2C,D). Of note, the *Smg5* knockout mESCs generated with this approach are pools of mESCs, and we designated them as ***Smg5*^iKO^** in our analysis.

Furthermore, in our study, we transiently expressed GFP or GFP-Cre expression vector in *Smg5*^F/F^CreER^T2-^ mESCs. Single GFP^+^ cells were sorted with FACS and seeded onto feeder layers to establish mESC colonies (Figure 2E). Transient expression of Cre recombinase will delete exons 2–4 of the *Smg5* allele. With this approach, we generated mESC colonies with SMG5 deletion. PCR on genomic DNAs, qPCR, and Western blotting analysis on samples from these single colonies further confirmed successful generation of *Smg5* knockout mESCs (Figure 2F–H). We designated these SMG5 knockout mESC lines as ***Smg5*^Δ/Δ^**.

**Figure 3:**
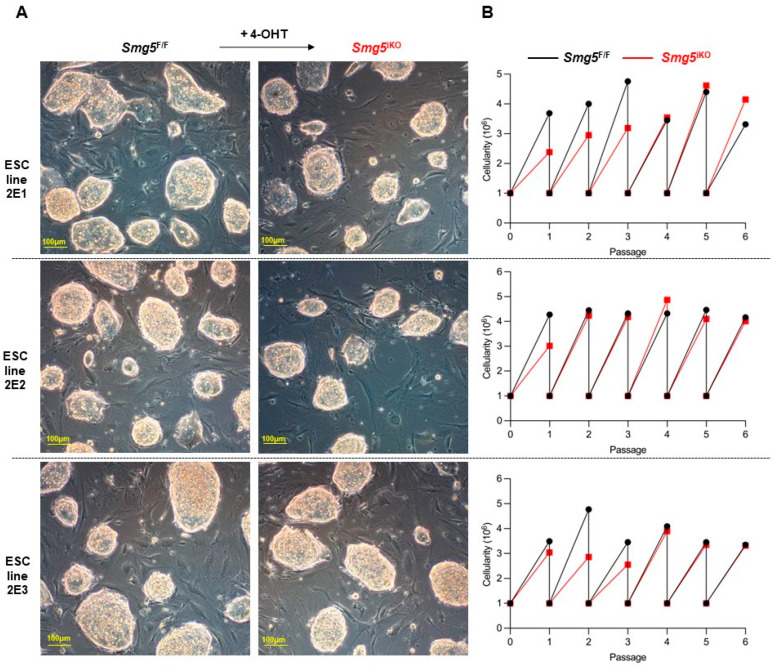
SMG5 is dispensable for mESC proliferation. (**A**) Morphology of control and *Smg5*^iKO^ mESCs on feeders. (**B**) Proliferation curve of control and *Smg5*^iKO^ mESCs (6 passages of these mESCs are investigated). Note: 2E1, 2E2, and 2E3 are the parental *Smg5* inducible knockout mESC lines.

Notably, the morphology showed no discernible differences between *Smg5*^F/F^ (control) and *Smg5*^iKO^ mESCs (Figure 3A), and the proliferation rate was comparable to that of *Smg5*^F/F^ (control) mESCs (Figure 3B). These findings were further verified with *Smg5*^Δ/Δ^ mESCs (Supplementary Figure S2A). Therefore, we conclude that SMG5 is dispensable for mESC proliferation.

### 3.3. SMG5 Deletion Inhibits NMD Activity in mESCs

**Figure 4:**
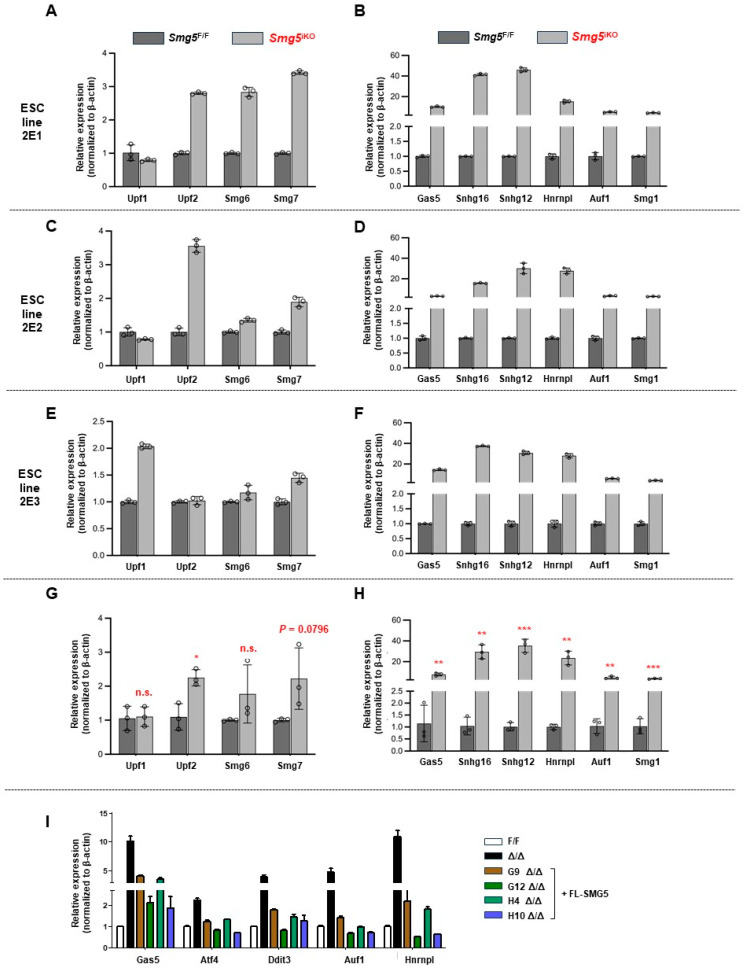
SMG5 is required for NMD activity in mESCs. (**A**) qPCR analysis on expressions of NMD factors (*Upf1*, *Upf2*, *Smg6*, and *Smg7*) in both control and *Smg5*^iKO^ mESCs (2E1 is the parental *Smg5* inducible knockout mESC line). Error bars represent the variance in technical replicates in qPCR assay. (**B**) qPCR analysis on expressions of NMD canonical targets (*Gas5*, *Snhg16*, *Snhg12*, *Hnrnpl*, *Auf1*, and *Smg1*) in control and *Smg5*^iKO^ mESCs (2E1 is the parental *Smg5* inducible knockout mESC line). Error bars represent the variance of technical replicates in qPCR assay. (**C**) qPCR analysis on expressions of NMD factors (*Upf1*, *Upf2*, *Smg6*, and *Smg7*) in both control and *Smg5*^iKO^ mESCs (2E2 is the parental *Smg5* inducible knockout mESC line). Error bars represent the variance in technical replicates in qPCR assay. (**D**) qPCR analysis on expressions of NMD canonical targets (*Gas5*, *Snhg16*, *Snhg12*, *Hnrnpl*, *Auf1*, and *Smg1*) in control and *Smg5*^iKO^ mESCs (2E2 is the parental *Smg5* inducible knockout mESC line). Error bars represent the variance in technical replicates in qPCR assay. (**E**) qPCR analysis on expressions of NMD factors (*Upf1*, *Upf2*, *Smg6*, and *Smg7*) in both control and *Smg5*^iKO^ mESCs (2E3 is the parental *Smg5* inducible knockout mESC line). Error bars represent the variance in technical replicates in qPCR assay. (**F**) qPCR analysis on expressions of NMD canonical targets (*Gas5*, *Snhg16*, *Snhg12*, *Hnrnpl*, *Auf1*, and *Smg1*) in control and *Smg5*^iKO^ mESCs (2E3 is the parental *Smg5* inducible knockout mESC line). Error bars represent the variance in technical replicates in qPCR assay. (**G**) qPCR analysis on expressions of NMD factors (*Upf1*, *Upf2*, *Smg6*, and *Smg7*) in both control and *Smg5*^iKO^ ESCs (data are summarized from 2E1, 2E2, and 2E3 mESCs). (**H**) qPCR analysis on expressions of NMD canonical targets (*Gas5*, *Snhg16*, *Snhg12*, *Hnrnpl*, *Auf1*, and *Smg1*) in control and *Smg5*^iKO^ mESCs (data are summarized from 2E1, 2E2, and 2E3 mESCs). (**I**) qPCR analysis on expressions of NMD targets in *Smg5*^Δ/Δ^ mESCs stably expressing GFP-SMG5 fusion protein. Note: unpaired Student’s *t*-test was carried out for statistical analysis. n.s., not significant; *, *p* < 0.05; **, *p* < 0.01; ***, *p* < 0.001.

To explore the impact of SMG5 null on NMD activity, we used qPCR to investigate the expressions of known NMD targets in control and ***Smg5*^iKO^** mESCs. mRNA transcripts of NMD factors are molecular targets of NMD [10,30]. We found a marked increase in the mRNA levels of *Upf1*, *Upf2*, *Smg6*, and *Smg7* in *Smg5*^iKO^ mESCs (Figure 4A,C,E,G). Western blotting analysis revealed upregulation in the protein expression of SMG6 and SMG7 in *Smg5*^iKO^ mESCs (Supplementary Figure S3A,B). In addition, gene transcripts for *Atf4*, *Ddit3*, *Gas5*, *Snhg12*, *Hnrnpl*, *Auf1*, and *Smg1* were significantly higher in *Smg5*^iKO^ mESCs (Figure 4B,D,F,H).

Alternative splicing events could generate mRNA isoforms containing PTCs, which can be stabilized and accumulated when NMD is inhibited. We further investigated the transcript levels of PTC^+^ isoforms resulting from exon skipping and exon inclusion events through RT-PCR. The PTC^+^ mRNA isoforms of *Hnrnphl*, *Trub2*, *Hnrnpk*, *Srsf6*, and *Ptbp2* generated by the exon skipping events showed a significant increase in *Smg5*^iKO^ mESCs (Supplementary Figure S3C). Additionally, PTC^+^ mRNA isoforms of *Pkm2*, *Tmem183*, *Srsf3*, and *Rps9* generated by exon inclusion events increased in *Smg5*^iKO^ mESCs (Supplementary Figure S3D).

To further confirm that the aforementioned NMD inhibition is attributed to SMG5 deletion, a plasmid expressing a fusion protein of full-length SMG5 and GFP (GFP-SMG5) was constructed and electroporated into *Smg5*-deficient mESCs. Four independent mESC colonies (G9, G12, H4, and H10, in our analysis) with stably expressed GFP-SMG5 were successfully established. qPCR was used to determine whether exogenous GFP-SMG5 expression can rescue the NMD defect in *Smg5*-deficient mESCs. We found that classical NMD targets, such as *Gas5*, *Atf4*, *Ddit3*, *Auf1*, and *Hnrnpl* were all deceased in these rescued mESC lines (Figure 4I). These results indicate that deletion of SMG5 inhibits NMD activities in mESCs.

### 3.4. SMG5-Null Compromises mESCs Differentiation

**Figure 5:**
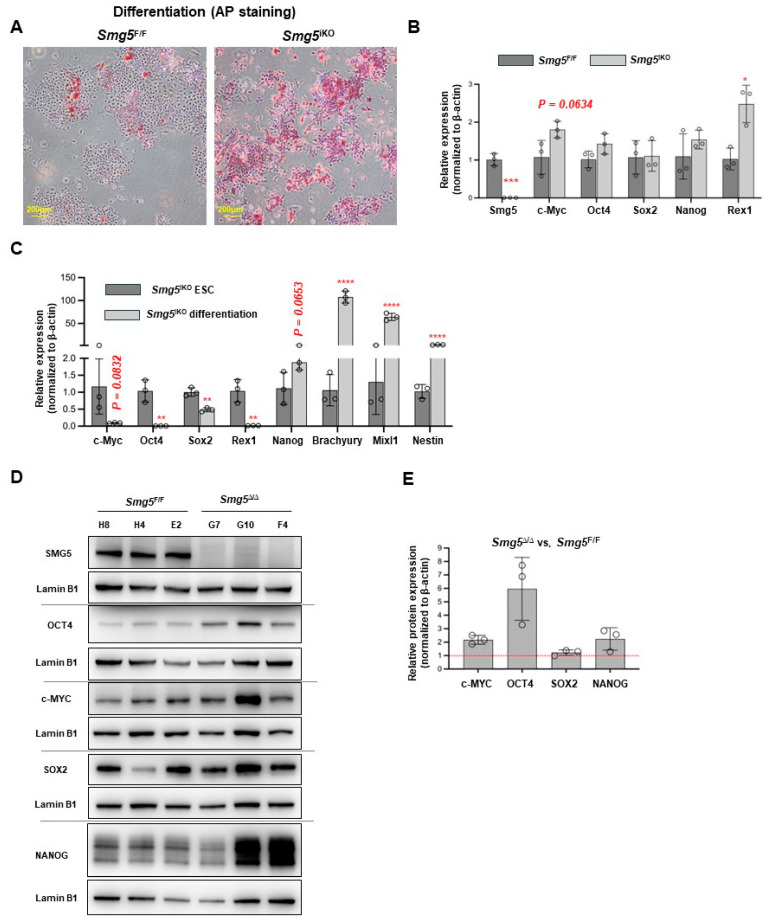
Deletion of SMG5 delays mESC differentiation. (**A**) Alkaline phosphatase (AP) staining on control and *Smg5*^iKO^ mESCs at differentiation day 4. (**B**) qPCR analysis on expressions of stemness markers in control and *Smg5*^iKO^ mESCs (day 4 under spontaneous differentiation condition); (**C**) qPCR analysis on expressions of stemness markers and lineage makers in *Smg5*^iKO^ mESCs and *Smg5*^iKO^ mESCs under spontaneous differentiation condition (day 4). (**D**) Western blotting analysis on expressions of stemness markers in control and *Smg5*^Δ/Δ^ mESCs under spontaneous differentiation condition (day 4). Lamin B1 was used as a loading control. (**E**) Quantification of the protein levels in control and *Smg5*^Δ/Δ^ mESCs under spontaneous differentiation condition (day 4). Unpaired Student’s *t*-test was carried out for statistical analysis. *, *p <* 0.05; **, *p <* 0.01; ***, *p <* 0.001; ****, *p <* 0.0001. Original images can be found in Supplementary Materials.

The fact that *Smg5*-deficient mESCs are viable while *Smg5* knockout mouse embryos die before E13.5 suggests that SMG5-null impairs mESC differentiation. To investigate this hypothesis, we performed the spontaneous differentiation assay with control and *Smg5*^iKO^ mESCs. After 4 days in the differentiation culture medium, *Smg5*^iKO^ mESCs showed stronger alkaline phosphatase (AP)-positive staining, whereas only small and scattered AP-positive colonies were seen in the control mESC cultures (Figure 5A). qPCR analysis revealed upregulation of stemness markers *c-Myc* and *Rex1* in *Smg5*^iKO^ mESCs under the spontaneous differentiation condition (Figure 5B). Notably, compared with *Smg5*^iKO^ mESCs in the ESC culture conditions, stemness markers *Oct4*, *Nanog*, and *Rex1* were downregulated, whereas differentiation markers *Brachyury*, *Mixl1*, and *Nestin* were upregulated in *Smg5*^iKO^ mESCs under the spontaneous differentiation condition (Figure 5C).

Similarly, Western blotting showed increased levels of c-MYC and OCT4 proteins in *Smg5*-deficient mESCs under the spontaneous differentiation condition (Figure 5D,E). Together, our data indicate that *Smg5*-null delays the mESC differentiation process.

### 3.5. SMG5 Modulates c-MYC Expression in mESCs Via Regulating Protein Synthesis

**Figure 6:**
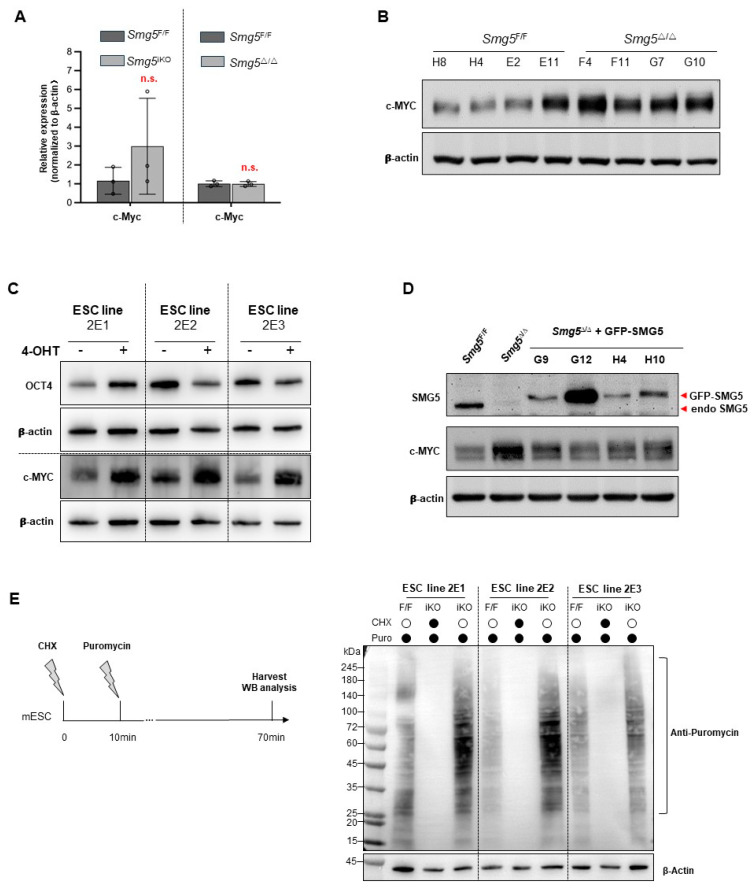
SMG5 regulates c-MYC expression. (**A**) qPCR analysis on the expression of *c-Myc* mRNAs in control, *Smg5*^iKO^, and *Smg5*^Δ/Δ^ mESCs. (**B**) Western blotting analysis on c-MYC protein expression in control and *Smg5*^Δ/Δ^ mESCs. (**C**) Western blotting analysis on expression of c-MYC proteins in control and *Smg5*^iKO^ ESCs. Note: 2E1, 2E2, and 2E3 are the parental *Smg5* inducible knockout mESC lines. (**D**) Western blotting analysis on c-MYC protein expression in distinct *Smg5*-deficient mESC clones expressing GFP-SMG5. An SMG5 antibody is used to detect the fusion proteins. (**E**) SUnSET assay in control and *Smg5*^iKO^ ESCs. Global protein synthesis (indicated by puromycin incorporation) was revealed by the anti-puromycin antibody staining on control and *Smg5*^iKO^ mESC protein samples. β-actin is used as a loading control for Western blotting. Note: unpaired Student’s *t*-test was carried out for statistical analysis; n.s., not significantly. Original images can be found in Supplementary Materials.

Depletion of SMG6 in mESCs leads to an elevation in *c-Myc* mRNA level, mediating the differentiation failure of *Smg6*-null mESCs [10]. We found that *c-Myc* mRNA and proteins are upregulated in *Smg5*-deficient mESCs under the differentiation condition. To test whether and how SMG5 relates to c-MYC expression in the ESC culture condition, we used qPCR and WB to investigate the expression of c-MYC. To our surprise, the mRNA levels of *c-Myc* in both *Smg5*^iKO^ and *Smg5*^Δ/Δ^ mESCs were comparable to those of the controls (Figure 6A). However, Western blotting revealed that protein expressions of c-MYC were significantly increased in *Smg5*^iKO^ and *Smg5*^Δ/Δ^ mESCs as compared with those of the controls (Figure 6B,C). Meanwhile, the c-MYC protein levels were effectively reduced when we stably expressed GFP-SMG5 proteins in *Smg5*^Δ/Δ^ mESCs (Figure 6D), suggesting that SMG5 does regulate c-MYC protein expression.

To verify whether SMG5 deletion increases protein translation, specifically c-MYC protein synthesis, we then performed a surface sensing of translation (SUnSET) assay (Figure 6E). Puromycin will be incorporated into newly synthesized poly-peptides. Using a puromycin-specific antibody, we found puromycin incorporation is increased in *Smg5*^iKO^ mESCs compared to the control (Figure 6E). This finding suggests that deletion of SMG5 enhances global protein synthesis.

### 3.6. SMG5 Loss Results in Abnormal Alternative Splicing Events Related to Cell Fate Transitions

**Figure 7:**
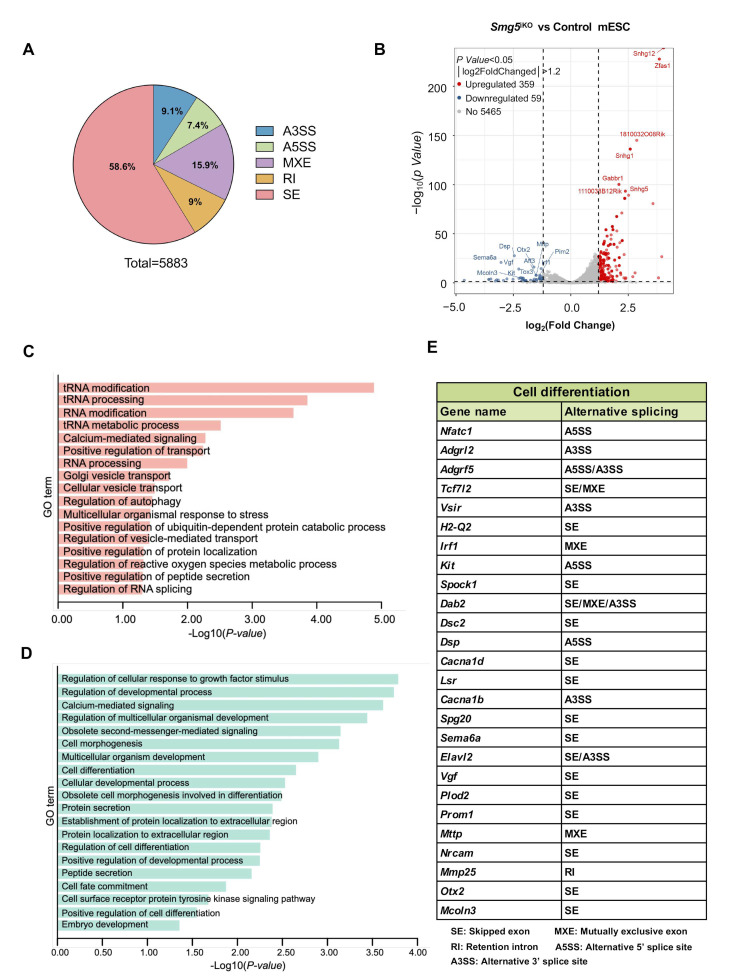
Deletion of SMG5 results in abnormal alternative splicing related to cell fate transitions. (**A**) Summary on alternative splicing events in control and *Smg5*^iKO^ mESCs. (**B**) Volcano map displaying differentially expressed genes with alternative splicing events in control and *Smg5*^iKO^ mESCs. A total of 418 differentially expressed gene transcripts were found. Among them, 359 gene transcripts were upregulated and 59 gene transcripts were downregulated (|log2FoldChanged| >1.2, *p* value < 0.05). (**C**) Gene ontology (GO) cluster analysis of significantly upregulated AS gene transcripts in control and *Smg5*^iKO^ mESCs. (**D**) Gene ontology (GO) cluster analysis of significantly downregulated AS gene transcripts in control and *Smg5*^iKO^ mESCs. (**E**) Abnormal alternative splicing events were detected in genes associated with cell differentiation.

To investigate additional molecular mechanisms of SMG5 in mESC cell fate determination and differentiation, we performed RNA-seq using control and *Smg5*^iKO^ mESCs (Supplementary Figure S4A). SMG5 deletion resulted in highly reproducible transcriptomic changes in mESCs, with 900 genes showing significantly upregulated expression and 736 genes showing significantly downregulated expression (|log2FoldChanged| > 1, *p* value < 0.05) (Supplementary Figure S4B). Since NMD has been linked to alternative splicing during development [24], we analyzed the RNA-seq data and found a significant number of abnormal alternative splicing events in the *Smg5*^iKO^ mESCs (Figure 7A), which included skipped exons (SEs) (58.6%), mutually exclusive exons (MXEs) (15.9%), alternative 5′ splice sites (A5SSs) (9.1%), retention introns (RIs) (9%), and alternative 3′ splice sites (A3SSs) (7.4%) (Figure 7A). Among these AS transcripts, 359 transcripts were upregulated and 59 transcripts were downregulated (Figure 7B). Gene ontology (GO) cluster analysis revealed that upregulated AS transcripts were clustered in the protein transport pathway and protein degradation pathway (Figure 7C), while downregulated AS transcripts were clustered in cell fate commitment, such as cell differentiation and development-related pathways (Figure 7D). Notably, compared with control mESCs, transcripts associated with the cell differentiation process in *Smg5*^iKO^ mESCs showed abnormal alternative splicing (Figure 7E). These findings further suggest that SMG5 may influence mESC cell fate by regulating alternative splicing.

## 4. Discussion

Nonsense-mediated mRNA decay (NMD) is an important mechanism of post-transcriptional regulation of gene expression in eukaryotes [11,12,13]. This process is responsible for identifying and degrading aberrant mRNAs (containing PTCs), thereby maintaining the quality of the transcriptome. Additionally, NMD can modulate the mRNA stability of gene transcripts coding proteins with physiological functions and control their protein production. In eukaryotic species, NMD is dispensable for life of yeasts, worms, and flies, while essential for the embryonic development of vertebrate animals [31]. NMD mutant mice die between E3.5 and E12.5, during which epiblasts differentiate into three germ layers and finally develop into different organs [8]. Thus, NMD is proposed to be essential for cell fate determination and cell fate transition during early embryonic development [8,9]. With SMG5 KO, SMG6 KO, and SMG7 KO ESCs generated with CRISPR-Cas9 technology, Huth et al. found the PTC isoforms of EIF4A2 contributed to the differentiation failure of SMG5 KO, while differentiation defects of SMG6 KO and SMG7 KO ESCs could be attributed to other undefined mechanisms [9]. By an inducible knockout approach, Li et al. found SMG6-null ESCs failed to differentiate due to upregulated of *c-Myc* [10]. Thus, the molecular mechanism mediated by different NMD factors in these processes is not well-defined.

In the current study, we generated an *Smg5* conditional knockout mouse line. By crossing the *Smg5* conditional knockout mouse with the Nestin-Cre transgenic mouse, we produced an *Smg5* conventional knockout mouse. *Smg5* knockout mice are embryonic lethal, and no viable embryos could be detected at E13.5. This finding indicated that NMD factor SMG5 is vital for embryonic development. Through crossing the *Smg5* conditional knockout mouse with the Cre-ER^T2+^ transgenic mouse, we produced an *Smg5* inducible knockout mouse line. We are able to generate multiple lines (at least seven different lines) of *Smg5* knockout mESCs. These SMG5-null mESCs are viable and show comparable proliferation as compared with their controls, although the expressions of some pluripotency markers are altered (for example, upregulated c-MYC protein in *Smg5*-null mESCs). The main defect of *Smg5*-null mESCs is the compromised differentiation, as shown in other studies on NMD factor knockout ESCs [9,10]. However, our study revealed that compared with control mESCs, the differentiation status of *Smg5*-null mESCs is delayed rather than blocked. Since null mutations of most NMD factors, such as SMG6, UPF1, UPF2, and UPF3A, die between the developmental time window of E5.5-E9.5 [8,32,33], it is highly plausible that NMD deficiency mESCs could still develop to the stage of organogenesis. Future studies concentrating on fine dissecting the embryonic lethality between E3.5-E9.5 will reveal more evidence on the developmental defects of NMD factor mutants.

Why do *Smg5*-null mESCs fail to differentiate properly? One reason is SMG5-mediated NMD activity [13,16]. All SMG5 KO lines in our studies showed NMD inhibition, as evidenced by upregulated transcripts of all known NMD targets, such as *Atf4*, *Ddit3*, *Gas5*, *Snhg12*, *Hnrnpl*, *Auf1*, and *Smg1*. With *Smg5* knockout mESCs generated with CRISPR-Cas9 technology, Huth et al. found that the PTC isoform of EIF4A2 is responsible for the differentiation defect [9]. In their experimental settings, they did not find that *c-Myc* mRNA and proteins are altered in 2i culture conditions. In our analysis, we used the feeder layer to support mESC growth and revealed that SMG5-null, not like SMG6 KO, has normal *c-Myc* mRNA expression, while the protein level of c-MYC is evidently increased. Intriguingly, when we ectopically expressed SMG5 in *Smg5*-deficient mESCs, the higher expression of NMD targets is rescued and c-MYC protein is negatively correlated with the protein levels of ectopically expressed SMG5. This finding indicates that SMG5 is required for c-MYC protein expression. Using the SUnSET assay, we found SMG5-null mESCs have higher global protein synthesis as compared with controls, which is further supported by our RNA-seq data. Our results suggested that enhanced protein synthesis of a certain pluripotency factor, such as c-MYC, may delay differentiation upon SMG5 loss.

Alternative splicing allows for a single gene locus to give rise to multiple mRNA isoforms, leading to proteomic diversity. mRNA isoforms with premature termination codons generated by alternative splicing will be degraded by NMD [31,34]. Furthermore, NMD deficiency could disturb the expression of splicing regulators, which will pose additional threat to global alternative splicing [34,35]. With our RNA-seq, we found SMG5-null mESCs show abnormal alternative splicing patterns as compared with control mESCs, which is consistent with findings conducted on *Upf2* and *Smg1* mouse mutants, where NMD inhibition was found to disturb AS during development [23,24,36]. We have identified a number of abnormally spliced genes in *Smg5*^iKO^ mESCs, which are related to cell fate commitment and developmental processes. The transcriptome data provide a rich resource for mining differentiation-related AS events.

## 5. Conclusions

In summary, in this study, we generated *Smg5* conditional knockout mice. To our knowledge, this current mouse line is the first *Smg5* CKO line, which will be useful in elucidating NMD biology. We found that SMG5 deletion is compatible with mESC viability and proliferation. Consistent with other NMD mutant ESCs, SMG5-null compromised ESC differentiation. Mechanistically, in our experimental settings (feeder-supported mESC growth), SMG5 may regulate c-MYC protein synthesis—and/or, alternatively, the splicing of cell differentiation gene transcripts—to safeguard mESC fate commitments.

## Data Availability

All datasets presented can be requested from the corresponding authors.

## Supplementary Materials

The following supporting information can be downloaded at: https://www.mdpi.com/article/10.3390/biom14081023/s1. Figure S1: Generation of *Smg5*^Δ/Δ^ mice and *Smg5* inducible knockout mESCs. Figure S2: Morphology of control (H8, H4, and E2) and *Smg5*^Δ/Δ^ (G7, G10, F4) mESCs growing on feeders. Figure S3: SMG5 loss inhibits NMD activity in mESCs. Figure S4: Transcriptome analysis between control and *Smg^5iKO^* mESCs.
