# Supplementary material for: RNA Surveillance Factor SMG5 Is Essential for Mouse Embryonic Stem Cell Differentiation
Source: Biomolecules. 2024 Aug 17;14(8):1023. doi: 10.3390/biom14081023 (PMC11352633; doi:10.3390/biom14081023)

Supplementary Figures

Supp Figure S1

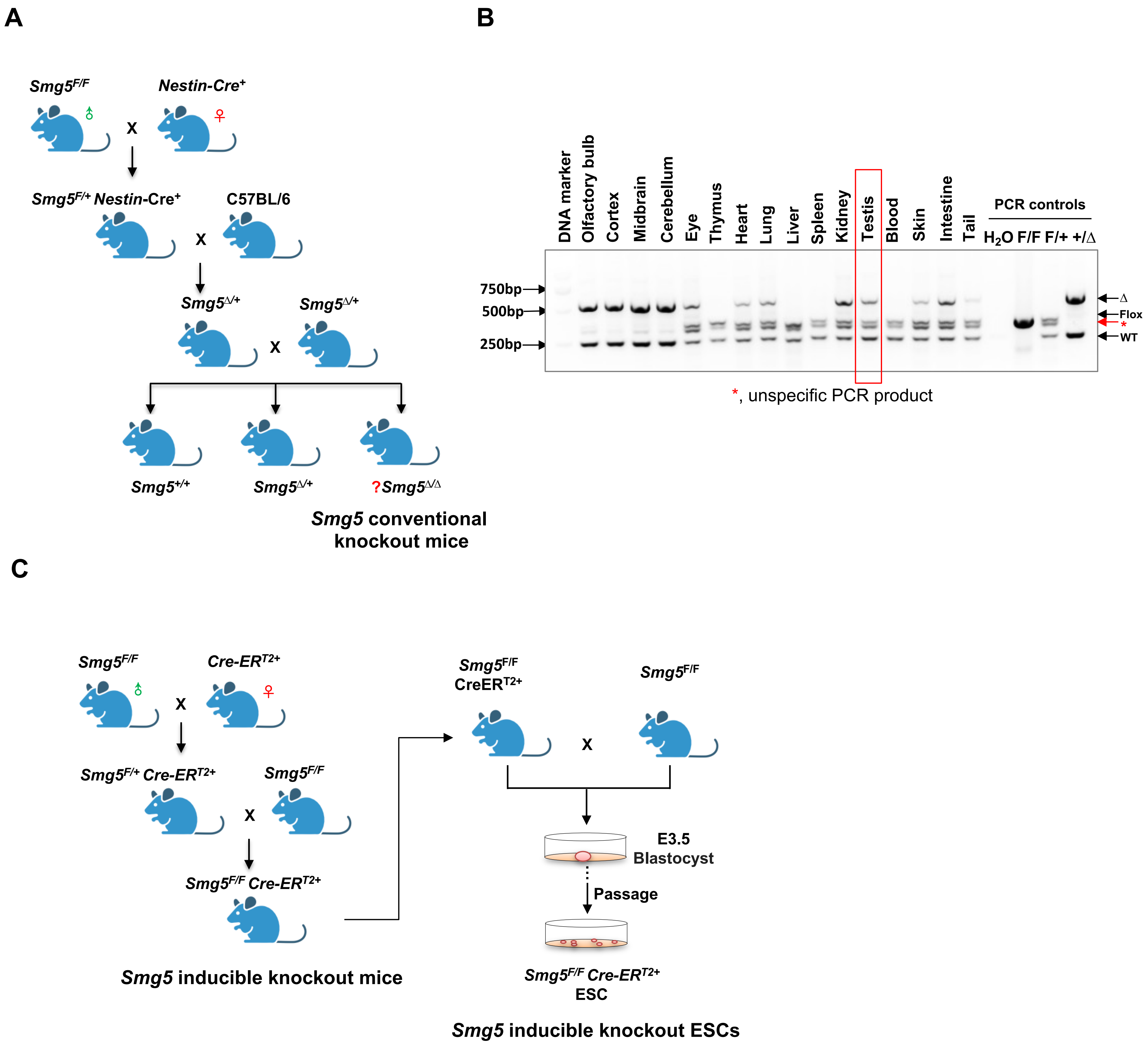

Supp Figure S1. Generation of *Smg5*$^{\Delta/\Delta}$ mice *and Smg5* inducible knockout mESCs. A. Breeding strategy to generate *Smg5*$^{\Delta/\Delta}$ mice. **B.** PCR analysis on *Smg5* deletion in various tissues from a male *Smg5*$^{F/F}$Nestin-Cre^{+} mouse. **C.** Strategy to establish ***Smg5*** inducible knockout mESCs (*Smg5*$^{F/F}$Cre-ER^{T2+} mESCs).

Supp Figure S2

A

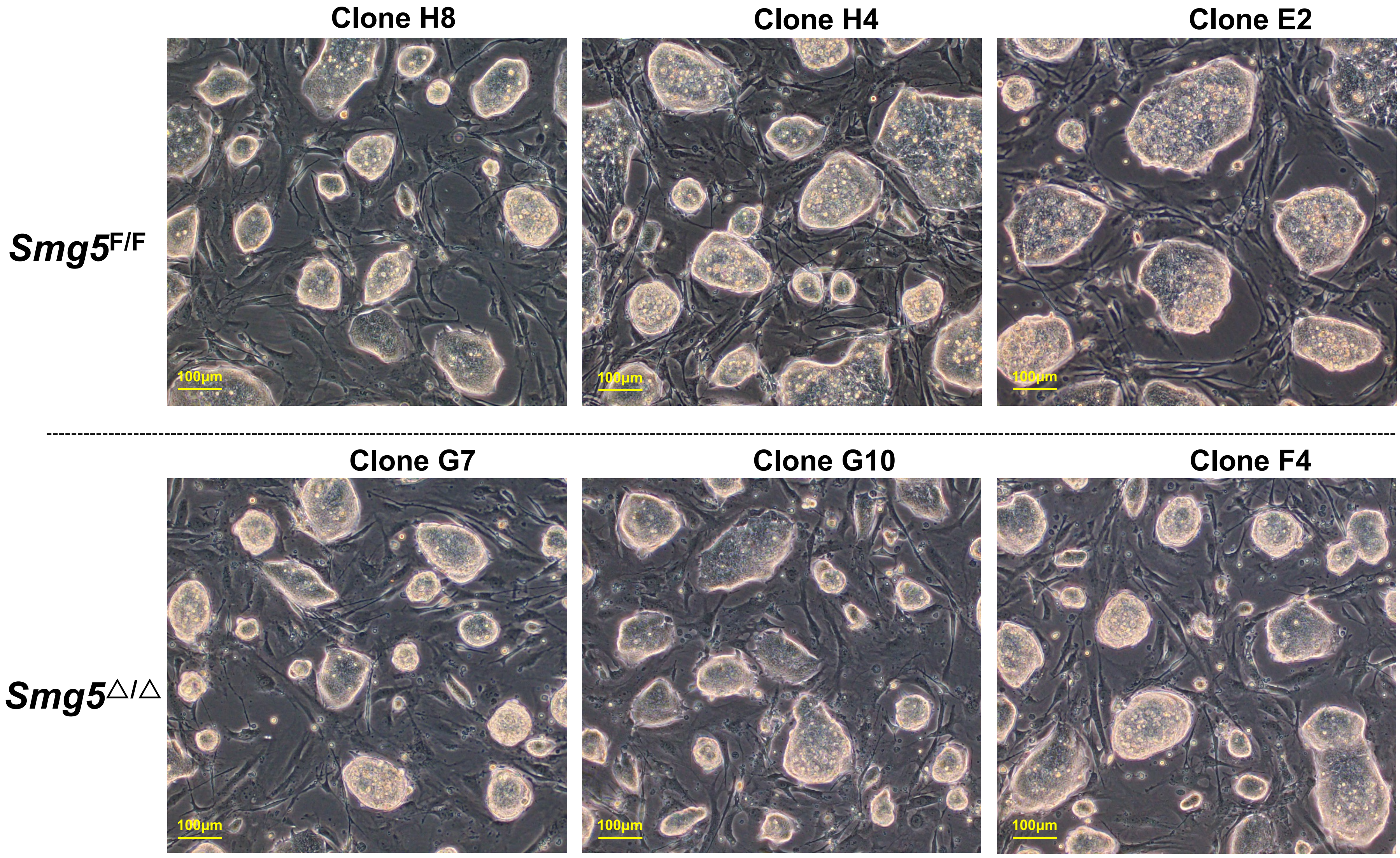

Supp Figure S2. Morphology of control (H8, H4, and E2) and *Smg5*$^{\Delta/\Delta}$ (G7, G10, F4) mESCs growing on feeders.

Supp Figure S3

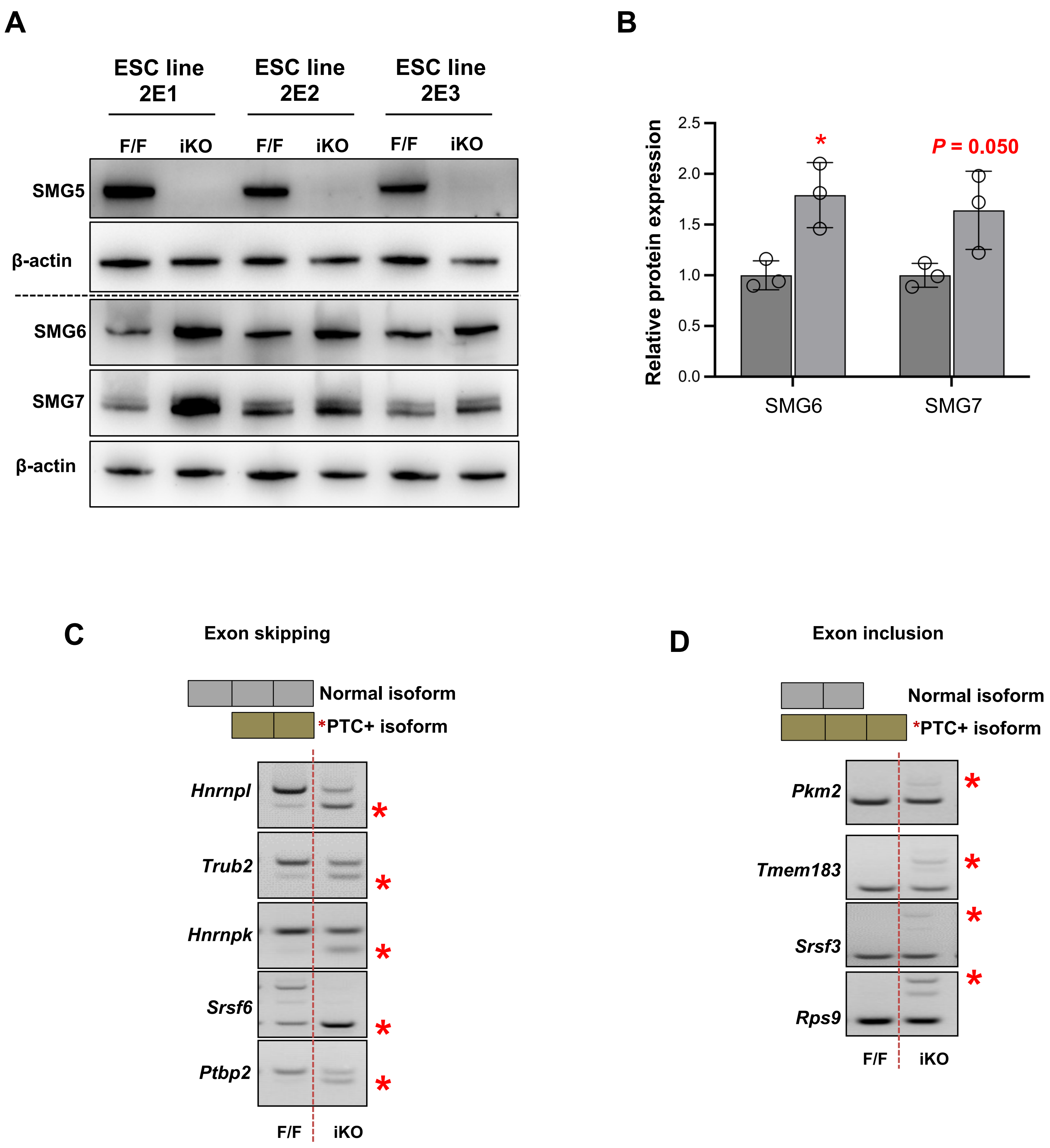

Supp Figure S3. SMG5 loss inhibits NMD activity in mESCs. A. Western blotting analysis of SMG5, SMG6 and SMG7 in control and *Smg5*^{iKO} mESCs. β-actin is used as a loading control. Note: 2E1, 2E2 and 2E3 are three parental *Smg5* inducible knockout mESC lines. **B.** Quantification of the protein levels of SMG6 and SMG7 in control and *Smg5*^{iKO} mESCs. **C.** Accumulation of PTC^{+} isoforms generated with exon skipping events in *Smg5*^{iKO} mESCs. **D.** Accumulation of the PTC^{+} isoforms generated with exon inclusion in *Smg5*^{iKO} mESCs. Note: Unpaired Student's *t*-test was carried out for statistical analysis. *, $p < 0.05$.

Supp Figure S4

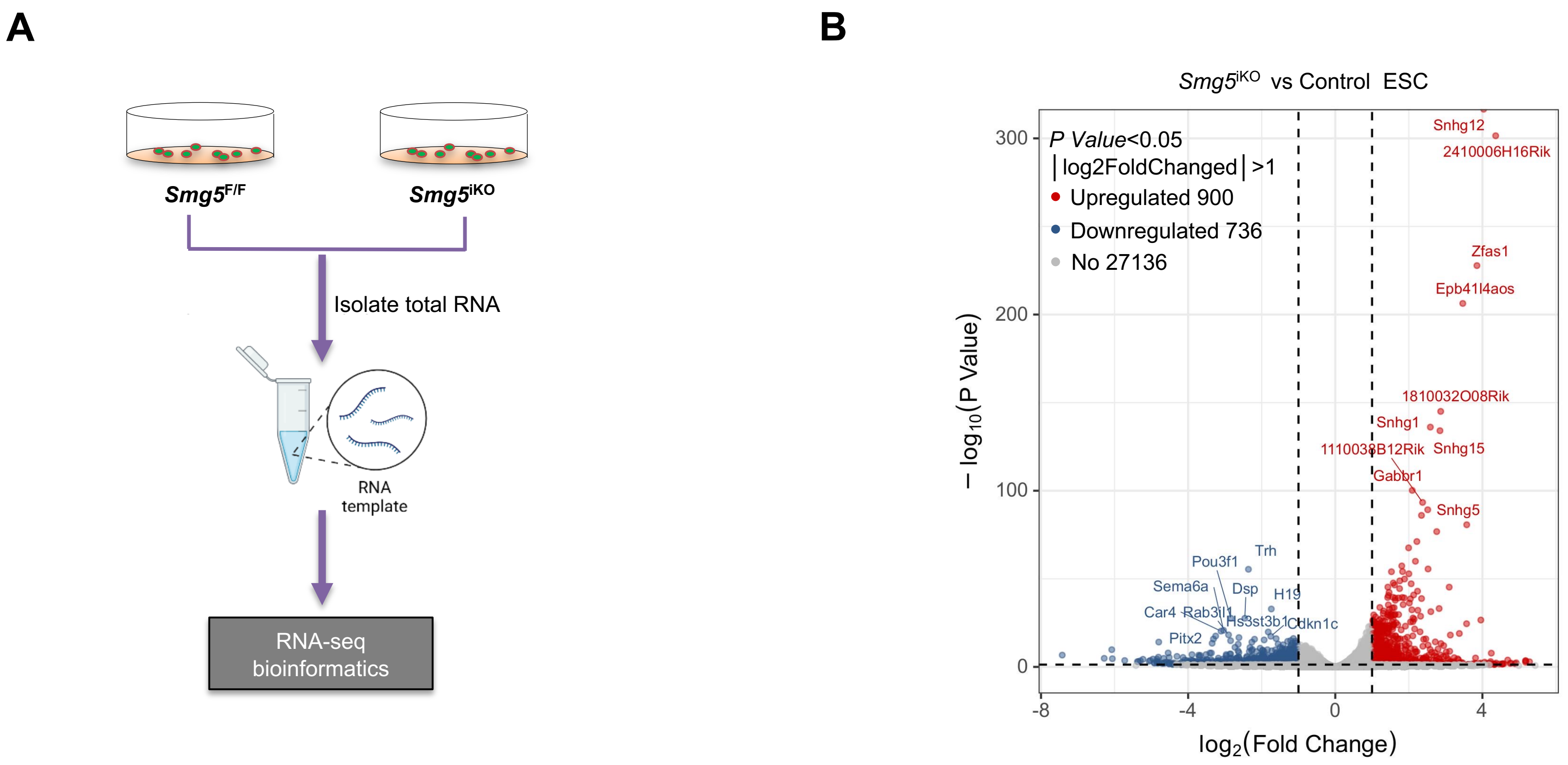

Supp Figure S4. Transcriptome analysis between control and *Smg5*^{iKO} mESCs. A. Schematic diagram of mESCs transcriptome sequencing. **B.** Volcano map displayed differentially expressed genes in control and *Smg5*^{iKO} mESCs. A total of 1636 differentially expressed genes were identified. Of these DEGs, 900 genes were upregulated, and 736 genes were downregulated (|log2FoldChanged| >1, *P Value*<0.05).

Original scans for main figures and supp figures

Related to Figure 2B

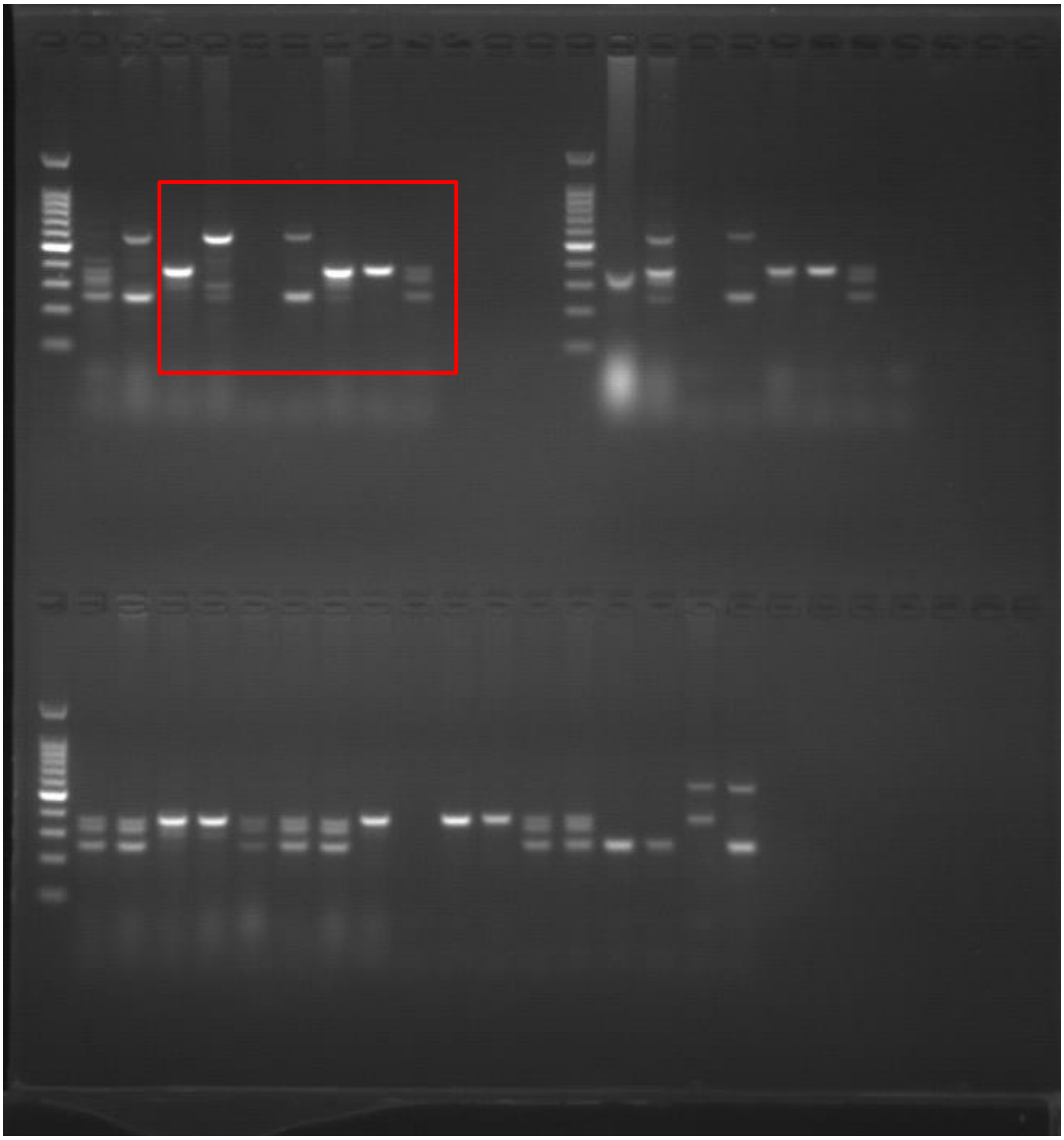

Related to Figure 2D

Smg5

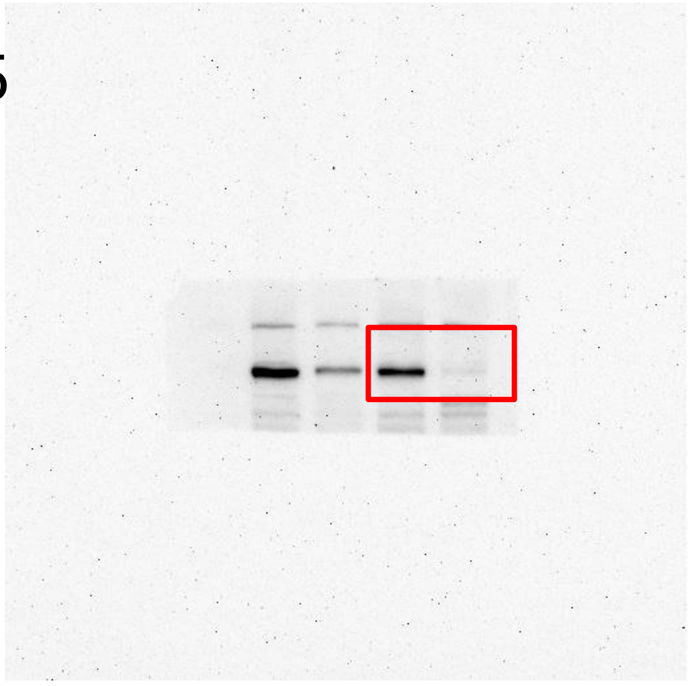

Actin

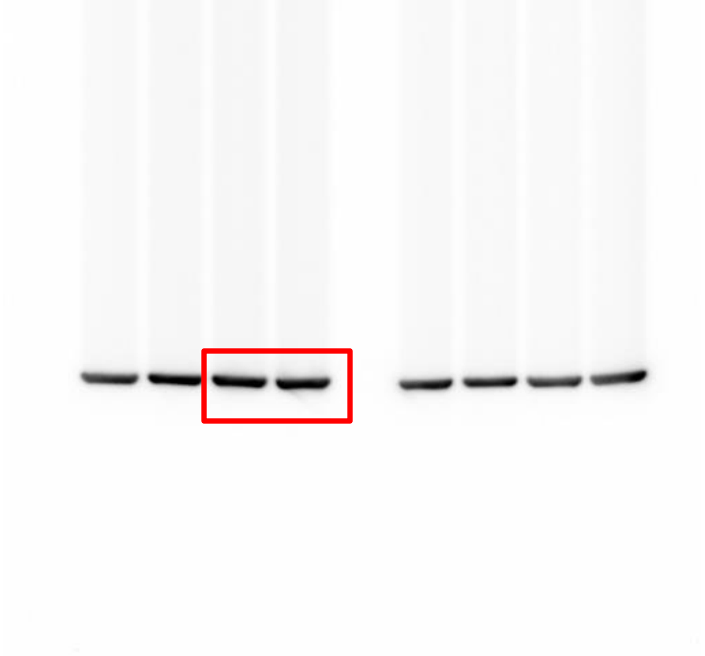

Related to Fig 2

Fig 2F

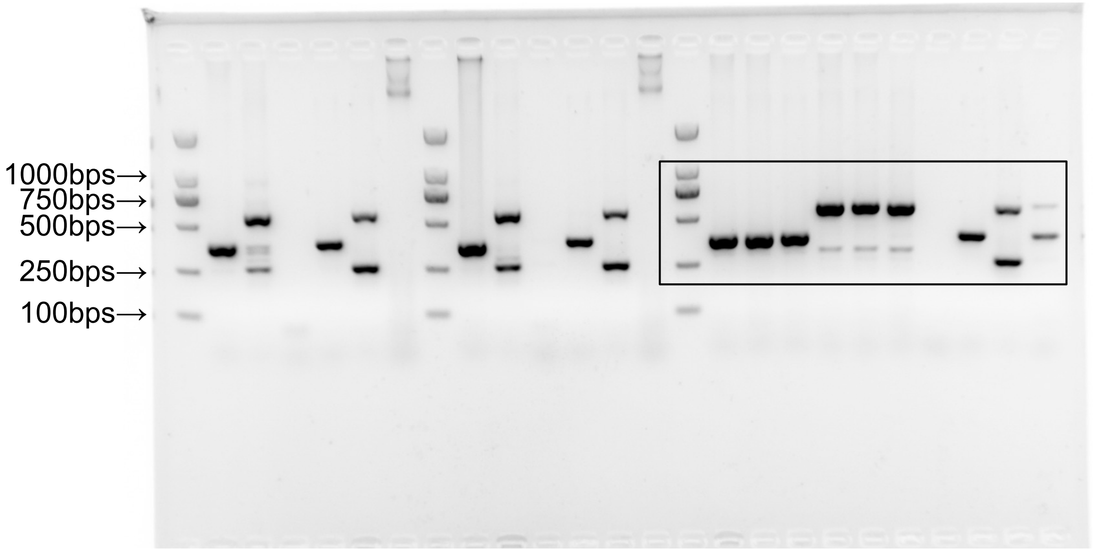

Fig 2H

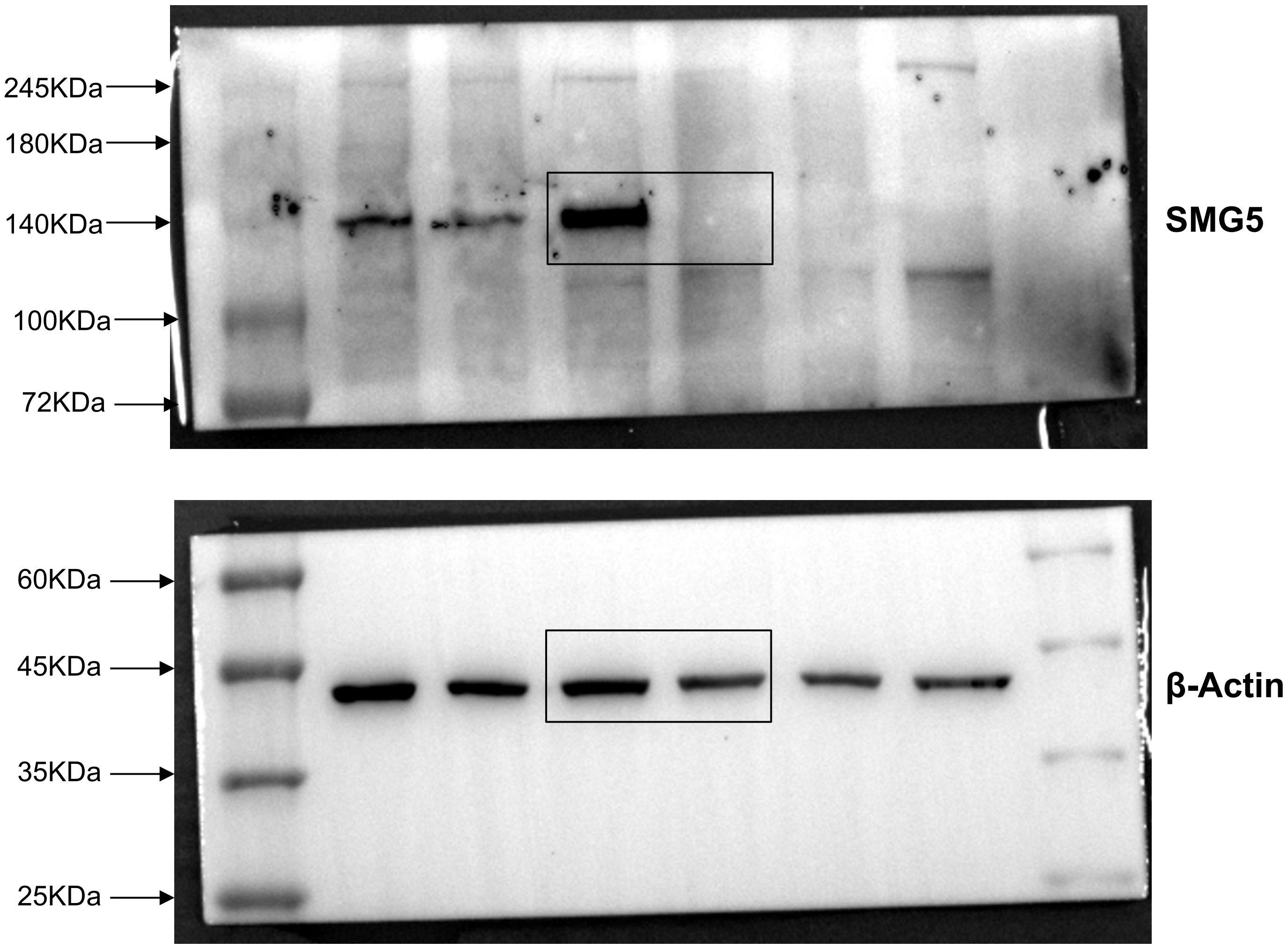

Related to Fig 5

Fig 5D

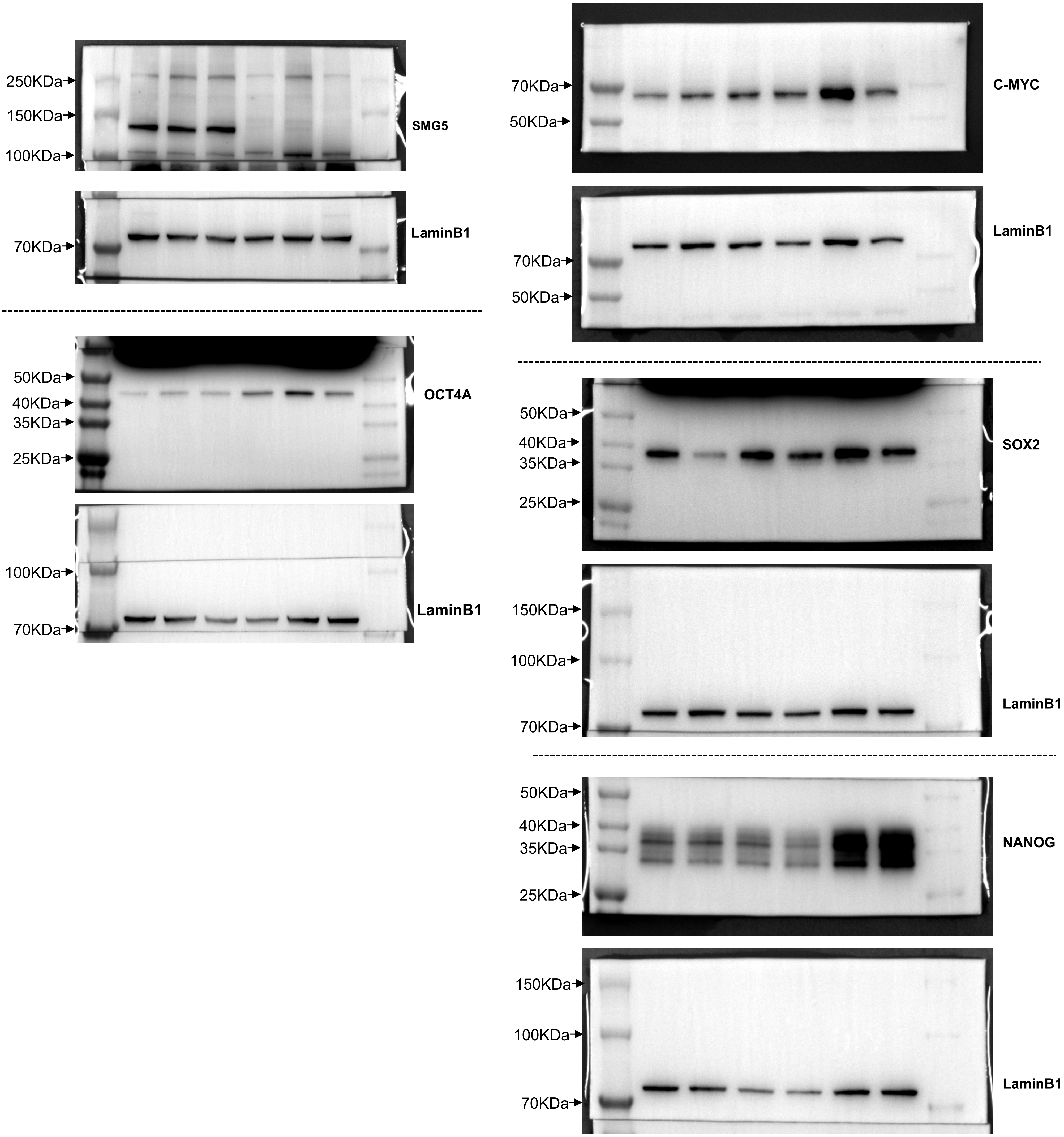

Related to Fig 6

Fig 6C

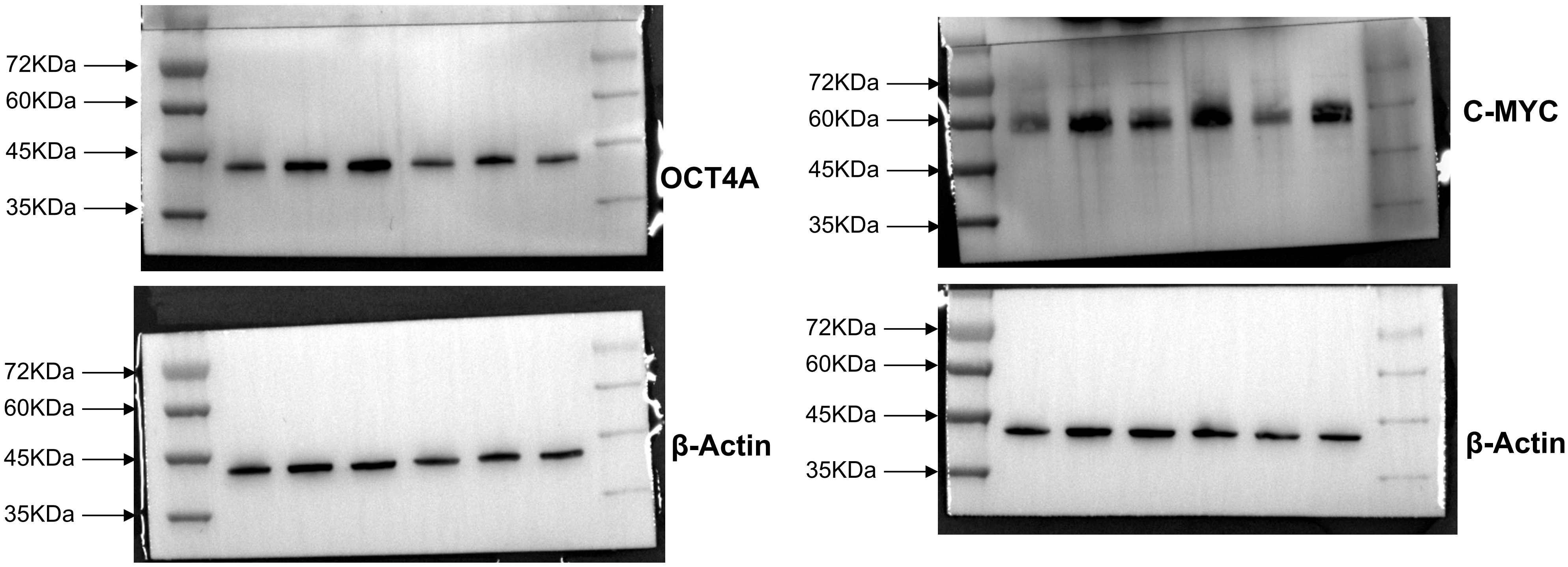

Fig 6E

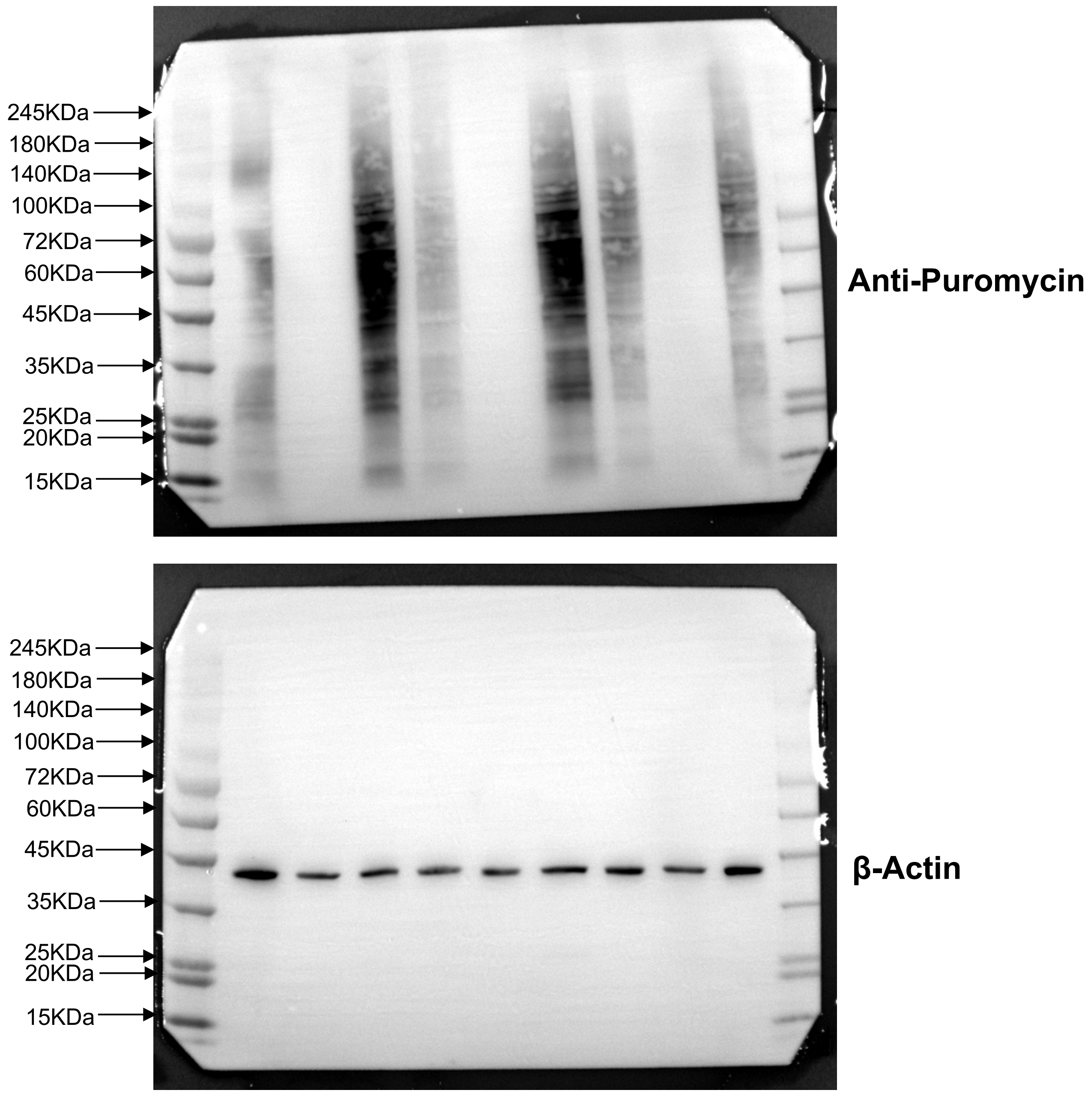

Related to Figure 6B

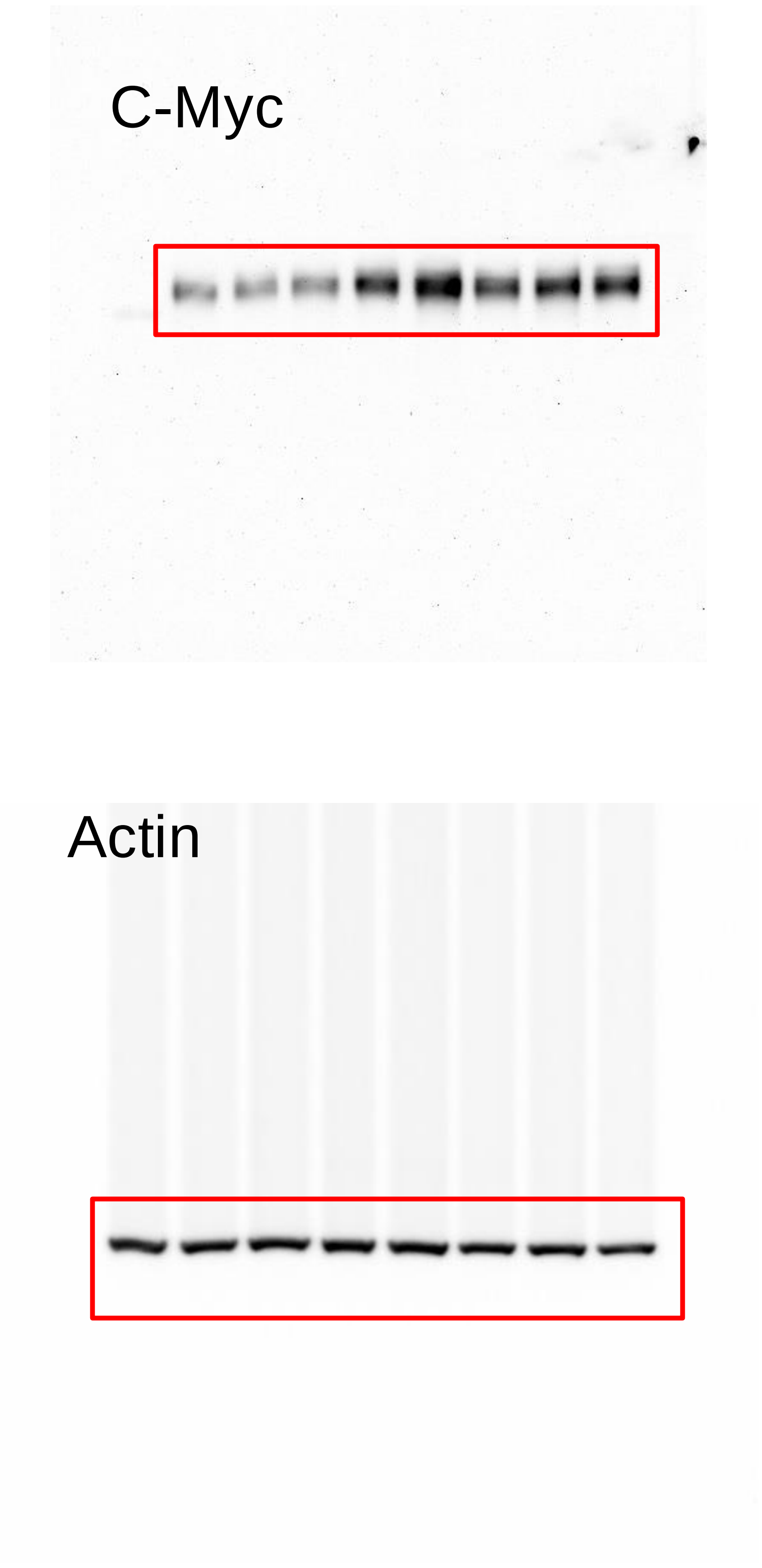

Related to Figure 6D

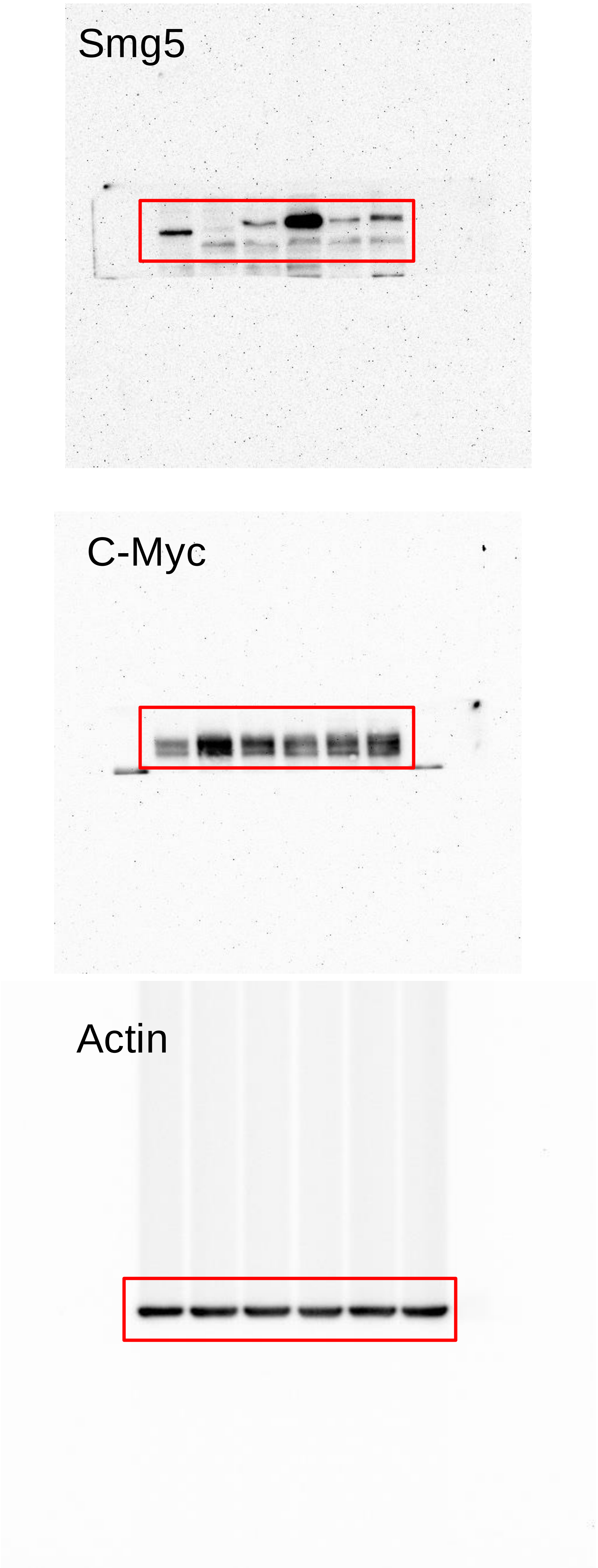

Related to Supp Figure1

Supp Figure 1B

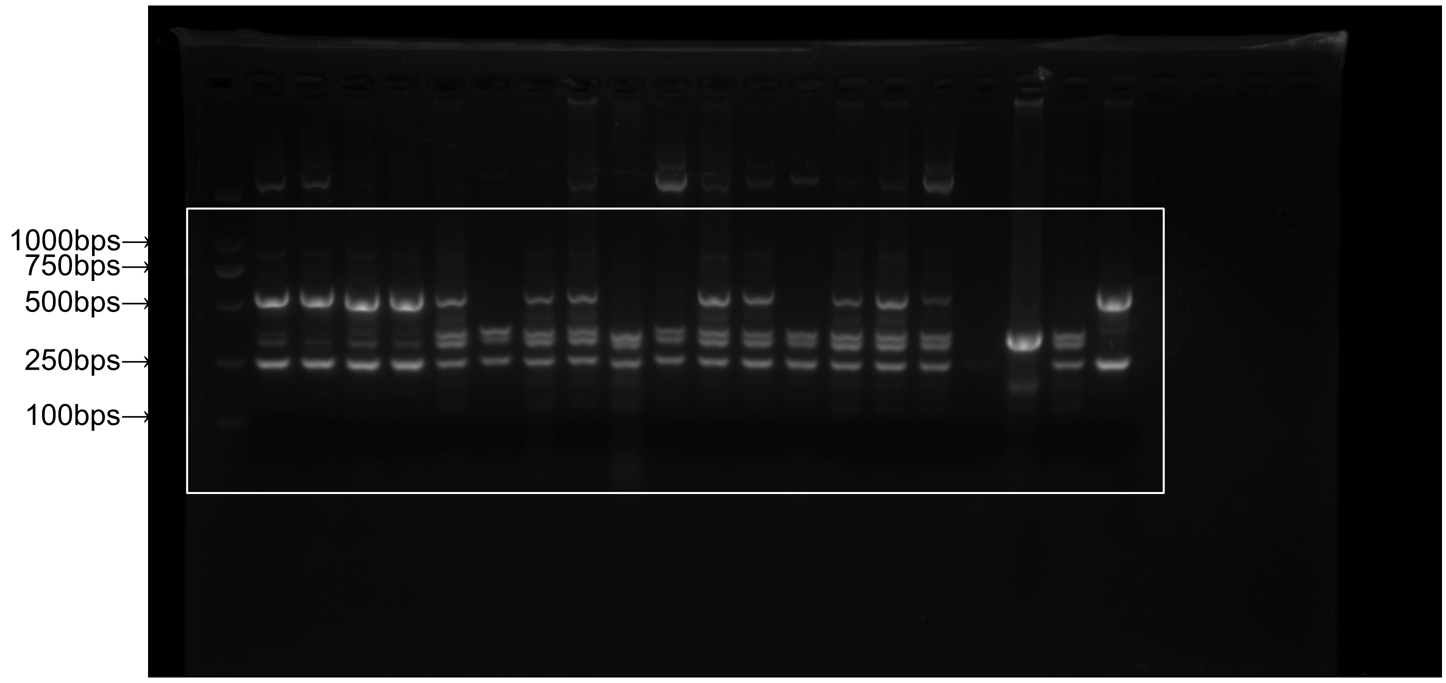

Related to Supp Figure3

Supp Figure 3A

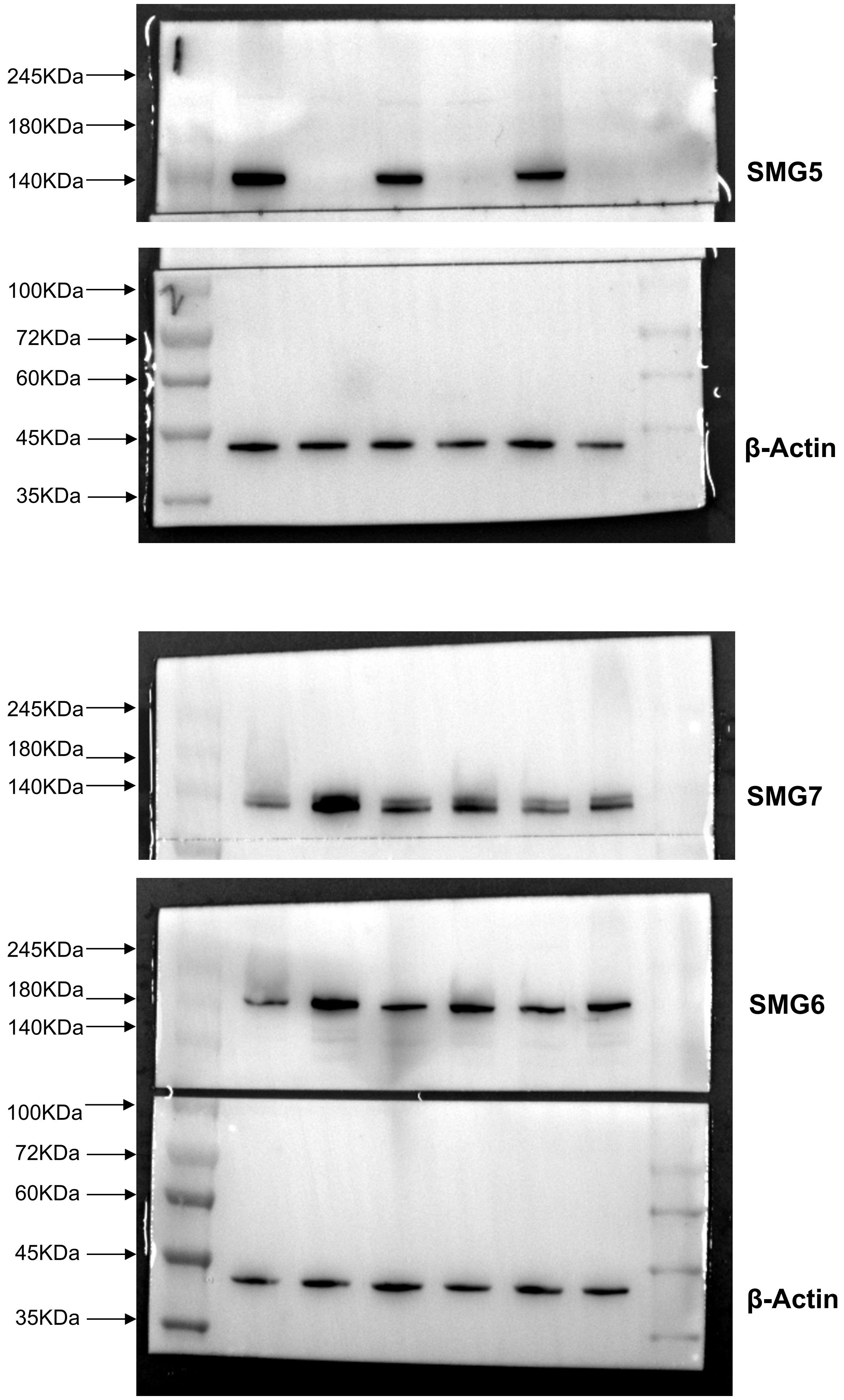

Related to Supp Figure 3C-D

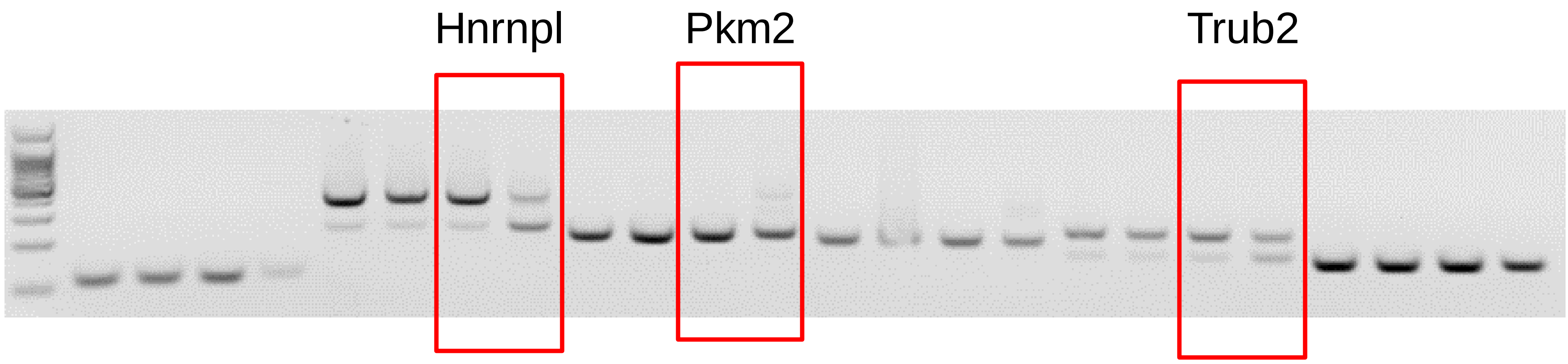

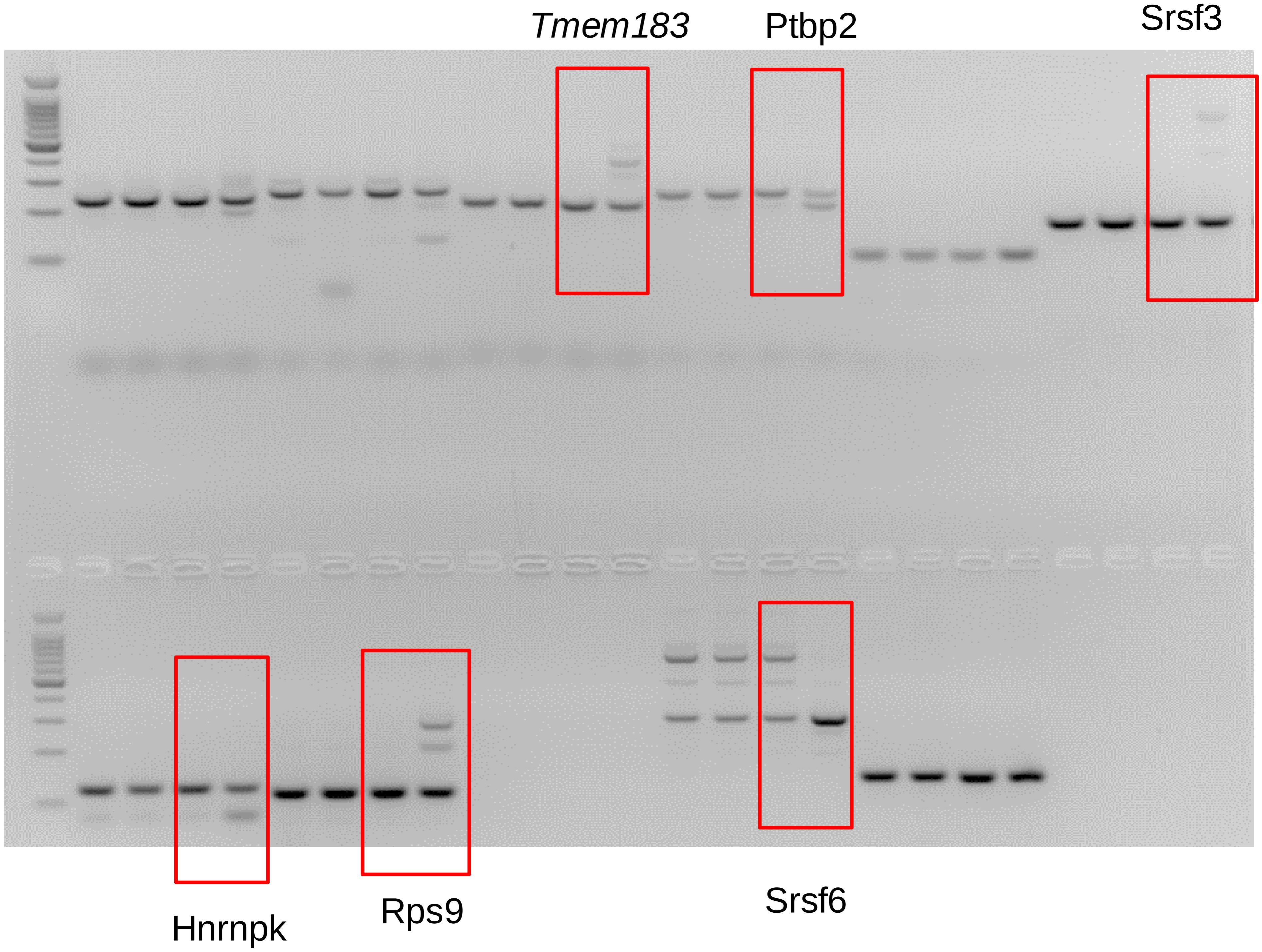

Supplement: Supplementary file 1 [file biomolecules-14-01023-s001.zip › biomolecules-3087275-supplementary.pdf]
